# Visible-Light-Driven Photocatalytic Hydrogen Production from Polystyrene Nanoplastics Using Pd/TiO_2_ Nanoparticles

**DOI:** 10.1021/acsanm.5c02376

**Published:** 2025-07-16

**Authors:** Angela Severino, Abdessamad Grirrane, María Cabrero-Antonino, Cristina Lavorato, Pietro Argurio, Raffaele Molinari, Hermenegildo García

**Affiliations:** † Instituto Universitario de Tecnología Química, 16774 Universitat Politecnica de Valencia-Consejo Superior de Investigaciones Científicas, Av. De los Naranjos s/n, 46022 Valencia , Spain; ‡ Department of Environmental Engineering (DIAm), 18950 University of Calabria, via P. Bucci, Cubo 44/A, 87036 Rende, CS, Italy

**Keywords:** nanomaterials, nanoplastics, polystyrene, photocatalysis, hydrogen production, photocatalytic degradation

## Abstract

The accumulation of microplastics and nanoplastics in aquatic environments has raised significant concerns in recent years, given the potential health risks to both aquatic ecosystems and humans; due to their nanometer size, they enter the food chain of aquatic species and consequently that of humans too. This study presents an efficient plasmonic photocatalyst for degrading polystyrene nanoplastics (PS NPs), while simultaneously generating green hydrogen in the process. Blank controls show that the presence of PS NPs is necessary for H_2_ evolution, since under identical conditions, it does not occur in their absence. A series of visible light-responsive plasmonic photocatalysts consisting of TiO_2_ nanoparticles (NPs) supporting Pd, Au, Pt, and Ag NPs were prepared via the impregnation method. Among the synthesized nanoparticle photocatalysts, the 3 wt % Pd/TiO_2_ NP photocatalyst exhibited superior hydrogen generation, producing 1329.76 μmol_H2_ g_cat_^–1^ after 2 h of irradiation, while also achieving a reduction in the average PS NP diameter. This study illustrates the potential of solar NP photocatalysis for environmental remediation and simultaneous hydrogen evolution.

## Graphical abstract

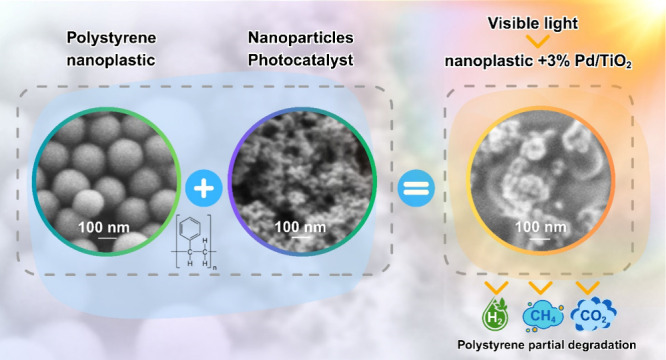

## 1 Introduction

Over the past 60 years, the use of plastics has grown exponentially due to their advantageous properties, including affordability, large-scale production, tunable mechanical resistance and inertness, ease of shaping, among many others.^1^ Most plastics are derived from petrochemical sources through optimized processes that have made monomer synthesis highly efficient and available at an industrial scale.^2^ However, improper management of plastic waste and its low biodegradability result in its persistent presence in the environment. It is estimated that 12 million plastic tons enter the ocean each year, with 9.5 million tons coming from land-based sources and 1.75 million tons directly coming from the fishing and shipping industries.^3^ Among plastic wastes, those that have raised increasing concerns are microplastics (MPs) and nanoplastics (NPs). MPs are classified as microparticles with sizes between 1 μm and 5 nm, while NPs are considered to be plastic materials with nanometric diameters. They are persistent pollutants that are produced either on purpose for certain applications such as cosmetics, toothpaste, medical products, and textile additives^4,5^ or by the physical abrasion in the environment of larger plastic items. MP and NP degradation occur at a very slow rate, and for this reason, they are considered as emerging, persistent contaminants.^6,7^

The presence of MPs and NPs in the environment has been widely documented in various studies.^8–12^ According to Hong et al.,^13^ MPs can be found in various shapes, including pellets, fragments, foams, fibers, spheres, and sheets. The most common polymer types identified in chemical analyses correspond to those plastics with larger production volume, such as polyethylene (PE), polypropylene (PP), polystyrene (PS), polyethylene terephthalate (PET), and polyvinyl chloride (PVC). Their stability, due to strong C–C bonds, makes them resistant to degradation and explains why they persist in the environment for extended periods.^14^

Numerous studies have highlighted the potential health risks associated with exposure to MPs and NPs, which include digestive and respiratory issues, endocrine disruption, metabolic changes, negative reproductive effects, among others.^14–16^ In particular, the unwanted health risks of polystyrene nanoplastics (PS NPs) have been studied in various research works. In one of them, Sökmen et al.^17^ microinjected polystyrene nanoplastics (PS NPs, 20 nm) into zebrafish embryos and found that the NPs accumulated in the brain, increasing mortality and abnormalities. Guimarães et al.^18^ reported that exposure to PS NPs altered the behavior of male Swiss mice, including locomotion, anxiety, and antipredator responses, and caused DNA damage in erythrocytes. Additionally, Li et al.^19^ observed that exposure to PS NPs constitutes a risk to fetal heart development in human embryonic stem cell-derived cardiomyocytes. Considering the risks of these plastic nanomaterials in the environment, among various technologies for reducing/eliminating pollution of MPs and NPs, such as coagulation, membrane filtration, and adsorption,^20,21^ photocatalytic technologies permitting upcycling into fuels and valorized chemicals are eco-friendly candidates.^20^ The use of photocatalytic nanomaterial particles is a promising approach for environmental remediation, and it has been used for both mineralization of plastic wastes and photoreforming.^22–24^ The mineralization, by degradation, of PS NPs in aqueous medium has been a matter of various studies,^20,25–28^ but photoreforming of microplastics is emerging as a dual-benefit technology, offering both environmental remediation and the generation of valuable products such as hydrogen and organic chemicals.^29,30^ This process uses photocatalysis in anaerobic conditions (hence different from mineralization, which is carried out in aerobic conditions) under solar or artificial light to break down microplastics in aqueous media and convert them into value-added chemical commodities. In particular, nanoplastics are used as sacrificial electron donors for degrading the unwanted polymeric pollutants, while concurrently producing hydrogen^31^ in a sustainable manner.^32^ Herein, we report the synthesis and testing of a visible light photocatalyst that efficiently generates hydrogen from PS NPs as a sacrificial agent.

Photocatalysis is considered an attractive technology to generate hydrogen, since it operates under ambient temperature and pressure and, eventually, it could use natural solar energy to drive the process.^33^ One recent strategy has been to couple photocatalytic hydrogen evolution with the simultaneous oxidative degradation of pollutants.^34–36^ The process relies on the development of efficient photocatalysts, such as metal oxide semiconductors, which frequently contain a cocatalyst to increase their efficiency.^37–39^ TiO_2_ is the most widely studied semiconductor for wastewater treatment^40^ and photocatalysis in general,^5,41^ due to its lack of toxicity, low cost, and chemical and photochemical stability.^42^

Despite its advantages, TiO_2_ suffers from two main limitations when used as a photocatalyst, i.e., the high recombination rate of photogenerated electron–hole pairs and the lack of photoresponse under visible light irradiation.^43^ Since UV light accounts for only a small portion (4%) of solar energy compared to visible light (43%), this UV-limited photoresponse results in poor efficiency for solar energy conversion.^44^ To overcome these limitations, plasmonic noble metal NPs, such as Au, Pt, Pd, Cu, and Ag, deposited on TiO_2_ NPs have been reported to diminish electron–hole pair recombination and extend light absorption into the visible region.^45,46^ In addition, the supported metals in plasmonic TiO_2_-based photocatalysts can also act as cocatalysts, favoring H_2_ evolution in photoreforming of organic molecules.^47–51^

Herein, we report an efficient visible light TiO_2_ photocatalyst that generates notable amounts of hydrogen using PS NPs as a sacrificial agent, thereby simultaneously causing some degradation of this noxious pollutant. Specifically, in this study, hydrogen generation from PS NPs (150 nm) is investigated using TiO_2_-based photocatalysts modified by supporting different noble metal NPs, including Pd, Au, Pt, and Ag, at various concentrations. The process can be considered pollutant photoreforming, since it is not observed in its absence, and PS NPs cause hydrogen evolution by their oxidation, rather than by H_2_O photoreduction.

## 2 Experimental Section

### 2.1 Chemicals

All chemicals were used directly as supplied, without additional purification. Milli-Q water was obtained using an IQ 7000 purifying system. Titanium dioxide (TiO_2_) P25 type (specific surface area: 44 m^2^ g^–1^; crystallographic phase: ca. 80% anatase and 20% rutile; 20 nm average primary particle size; band gap: 3.3 eV, as measured and reported in the Supporting Information) was purchased from Evonic–Degussa, and HAuCl_4_·3H_2_O was supplied by Alfa Aesar. The rest of the chemicals, H_2_PtCl_6_·H_2_O, Pd­(NO_3_)_2_·2H_2_O (99.9% trace metals basis), cerium­(IV) oxide (CeO_2_) nanopowder (<25 nm particle size (BET), 7.13 g mL^–1^ density), NaOH (reagent grade, 97%, powder, NaOH), 1-phenylethanol (97%), Pd/C catalysts containing 1 wt %Pd, and AgNO_3_ (63.5% purity) were provided by Sigma-Aldrich Products. The samples of monodispersed PS NPs used in the present study, having a nominal mean size of 150 nm and a standard deviation of 3 nm in a 5% (w/v) aqueous suspension, were supplied by Microparticles GmbH (Berlin, Germany).

### 2.2 Photocatalyst Preparation

The photocatalyst nanomaterials were prepared adapting procedures previously reported.^52,53^ Scheme 1 shows a schematic representation of the photocatalyst synthesis. Specifically, 1 wt % Pd/TiO_2_ and 1 wt % Pd/CeO_2_ photocatalysts were prepared using the same procedure. Pd/TiO_2_ photocatalysts were prepared at three different loadings by impregnating 250 mg of commercial TiO_2_ with 7 mL of Milli-Q water containing 5.41, 16.24, or 27.07 mg of Pd­(NO_3_)_2_·2H_2_O, corresponding, respectively, to samples with nominal values of 1, 3, or 5 wt % of Pd on the metal oxide assigned as labels. The resulting slurries were stirred for 2 h at room temperature, after which the water was evaporated, and the solids were dried overnight at 100 °C. The solids were then reduced using 1-phenylethanol at 160 °C for 2 h. Finally, the photocatalyst was washed, filtered, and dried at room temperature for 12 h. The amount of metal loaded on the photocatalyst nanoparticles was determined and discussed in Section 3.1.

**Scheme 1:**
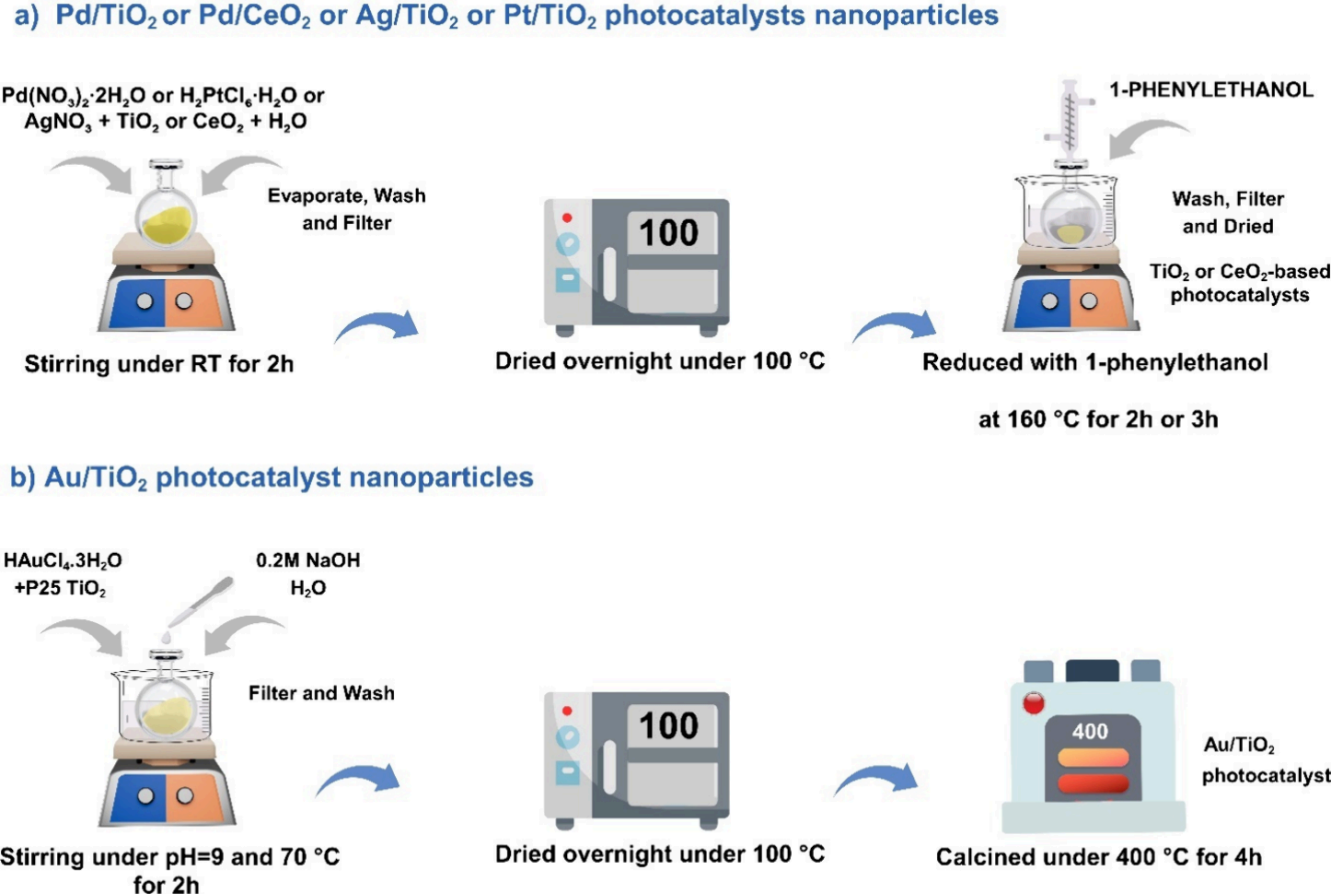
Schematic Representation of the Preparation of TiO_2_ or CeO_2_-Based Photocatalysts, (a) Pd/TiO_2_, Pd/CeO_2_, Ag/TiO_2_, and Pt/TiO_2_ Photocatalyst Nanoparticles and (b) Au/TiO_2_ Photocatalyst Nanoparticles

Au/TiO_2_ was prepared by the deposition–precipitation method, utilizing 300 mg of HAuCl_4_ and 10 g of TiO_2_ in deionized water (45 mL) to deposit Au onto TiO_2_, corresponding to a nominal value of 1 wt % Au on TiO_2_. The deposition procedure was carried out at 70 °C and pH of 9, using a 0.2 M aqueous NaOH solution to maintain constant pH values during the 2 h adsorption process. The Au/TiO_2_ photocatalyst was then recovered, filtered, washed with deionized water, and dried at 100 °C overnight. Finally, the powder was calcined at 300 °C in air for 4 h.

Pt/TiO_2_ was prepared by the impregnation method using 2 g of TiO_2_ that was suspended in 7 mL of Milli-Q water containing 56 mg of H_2_PtCl_6_·H_2_O, corresponding to a nominal value of 1 wt % of Pt on TiO_2_. The resulting slurry was stirred for 2 h at room temperature. After this time, the water was evaporated, and the solid was dried overnight at 100 °C. Pt on TiO_2_ was then reduced using 1-phenylethanol at 160 °C for 2 h. Finally, the Pt/TiO_2_ photocatalyst was washed, filtered, and dried at room temperature for 12 h.

Ag/TiO_2_ was prepared by the impregnation of 2 g of TiO_2_ with a solution of 0.03 g of AgNO_3_ in 10 mL of Milli-Q water. The slurry was stirred at room temperature for 2 h. After this time, the water was evaporated, and the solid was dried at 100 °C overnight. Ag on TiO_2_ was then chemically reduced by pouring the solid in 5 mL of 1-phenyletanol and heating the suspension at 160 °C for 3 h. Finally, the resulting Ag/TiO_2_ photocatalyst was washed, filtered, and dried at room temperature for 12 h.

### 2.3 Photocatalyst Characterization

Transmission electronic microscopy (TEM), EDX mapping, and EDX element line profiles were performed using a Philips CM300 FEG electron microscope with an accelerating voltage of 200 kV. The samples were first dispersed in ethanol and sonicated at 440 W for 15 min to obtain a homogeneous suspension. A drop of this suspension was deposited onto a Cu grid. X-ray diffraction (XRD) patterns were acquired with a Shimadzu XRD-7000 diffractometer by using Cu Kα radiation. The XRD patterns were collected in the 2θ range from 10 to 80° at a scan rate of 10° min^–1^. The actual metal loadings were determined by inductively coupled plasma atomic emission spectroscopy (ICP-AES) using a Varian 715-ES instrument elemental analyzer. Diffuse reflectance UV/vis spectra were recorded with a Varian Cary 5000 spectrophotometer in the wavelength range 200–800 nm. The samples were also analyzed by X-ray photoelectron spectroscopy (XPS) using a SPECS spectrometer operating with monochromatic Al Kα radiation. XPS data were calibrated taking the C 1s peak set at 284.5 eV, and the data were processed with the Casa XPS software. High-resolution XPS peaks were fitted by using XPSPEAK1 software after the Shirley baseline correction.

### 2.4 Photocatalytic Hydrogen Production Tests

Photocatalytic experiments were carried out using the output of a Xe lamp (150 W, Hamamatsu ref L8253; Hamamatsu spot light source L9566–04 and light guide A10014–50–0110, 100 mW cm^–2^) as the light source, filtering the beam with a cutoff filter that allows to pass only radiation of wavelengths longer than 450 nm. The photoreactor consisted of a cylindrical quartz vessel (51 mL total volume), with an inlet and outlet, each fitted with independent pressure gauges and a manometer to monitor pressure. To ensure the absence of oxygen in the system, the entire system was purged with an argon flow for at least 30 min prior to irradiation. The photocatalytic solution (20 mL), containing a suspension of 200 μg of PS NPs and various amounts of photocatalyst (10, 20, 30, and 40 mg) or different photocatalysts (1, 3, and 5 wt % Pd/TiO_2_, 1 wt % Pd/CeO_2_, 1 wt % Pd/C, 1 wt % Au/TiO_2_, 1 wt % Pt/TiO_2_, and 1 wt % Ag/TiO_2_), was continuously magnetically stirred during irradiation at ambient temperature.

### 2.5 Analysis of Hydrogen Generation and Polystyrene Degradation

Hydrogen evolution during irradiation was quantified by gas chromatography-GC (Agilent 490 MicroGC equipped with a thermal conductivity detector and two channels, one with a MolSieve 5 A column to analyze H_2_, O_2_, N_2_, and CO, while the other had a Pore Plot Q column to analyze CO_2_, CH_4_, and short-chain hydrocarbons). The temperature program was set from 50 to 280 °C, with a heating rate of 10 °C min^–1^. The moles of hydrogen generated were calculated from the GC data on H_2_ volume percentage, considering the gas phase as a mixture of ideal gases and applying the law of *n* = *PV*·*RT* ^–1^. The size distribution of the PS NPs at final time was determined with a Malvern Panalytical Zetasizer instrument. Adsorption of the photocatalyst on PS NPs during the reaction was confirmed by a Malvern Panalytical zeta potential instrument and FE-SEM imaging of a micro drop of the suspension, using a scanning electron microscope (TESCAN MIRA3, Czech Republic).

## 3 Results and Discussion

### 3.1 Photocatalyst Characterization

Photocatalytic H_2_ evolution from aqueous PS NP suspensions was studied under visible light irradiation using a series of plasmonic photocatalysts based on nanoparticulate TiO_2_ oxide supporting metal nanoparticles of Pd, Au, Pt, and Ag. These four metals were supported on TiO_2_ and labeled as reported in Table 1. Pd was supported on three samples on TiO_2_ nanoparticles, and Pd NPs were also deposited at 1 wt % on CeO_2_ and active carbon to determine the support role in the process. The samples were prepared by impregnating the required amount of the noble metal precursor on any of the two metal oxides, followed by a reduction step performed either thermally (Au/TiO_2_) or chemically using 1-phenylethanol at 150 °C as the reducing agent, as described in Section 2.2. The exact noble metal loading on the metal oxide semiconductor was determined by digesting the solid with *aqua regia* analysis of the resulting liquor using ICP-AES elemental analysis. The results are presented in Table 1.

**Table 1:** Photocatalysts Label and Experimental Metal Loading on the Series of Photocatalysts under Study

photocatalyst	experimental metal loading (wt %)
1 wt % Pd/TiO_2_	0.65
3 wt % Pd/TiO_2_	2.51
5 wt % Pd/TiO_2_	4.06
1 wt % Au/TiO_2_	0.51
1 wt % Pt/TiO_2_	1.23
1 wt % Ag/TiO_2_	0.89
1 wt % Pd/CeO_2_	0.61
1 wt % Pd/C	0.57

Pd NP size distribution and average particle size were determined by TEM measurements. As an example, Figure 1 shows high-resolution TEM images of several photocatalysts prepared in this study to illustrate these measurements. The average metal particle size distribution on the photocatalysts is reported in the Supporting Information: Pd (Figure S1), Au (Figure S2), Pt (Figure S3), and Ag (Figure S4). TiO_2_ was approximately 20 nm, and this size did not change upon deposition of each of the noble metals. The presence of noble metals, all heavier than Ti, was assessed by STEM-EDX and SEM-EDX analysis for all photocatalysts (Figures S5–S8) and was also confirmed by TEM images, in which they appear as smaller, higher-contrast, darker spots with an average size of about 5 nm for Pt/TiO_2_ (Figure 1c). In the cases of Pd/TiO_2_ (Figure 1a), Au/TiO_2_ (Figure 1b), and Ag/TiO_2_ (Figure 1d), the lattice fringes measured by high-resolution TEM were 2.1, 2.4, and 1.7 Å, respectively, that agree well with the reported values for the (111) facet of metallic Au and Pd,^53^ and the (200) facet of metallic Ag.^54^

**Figure 1:**
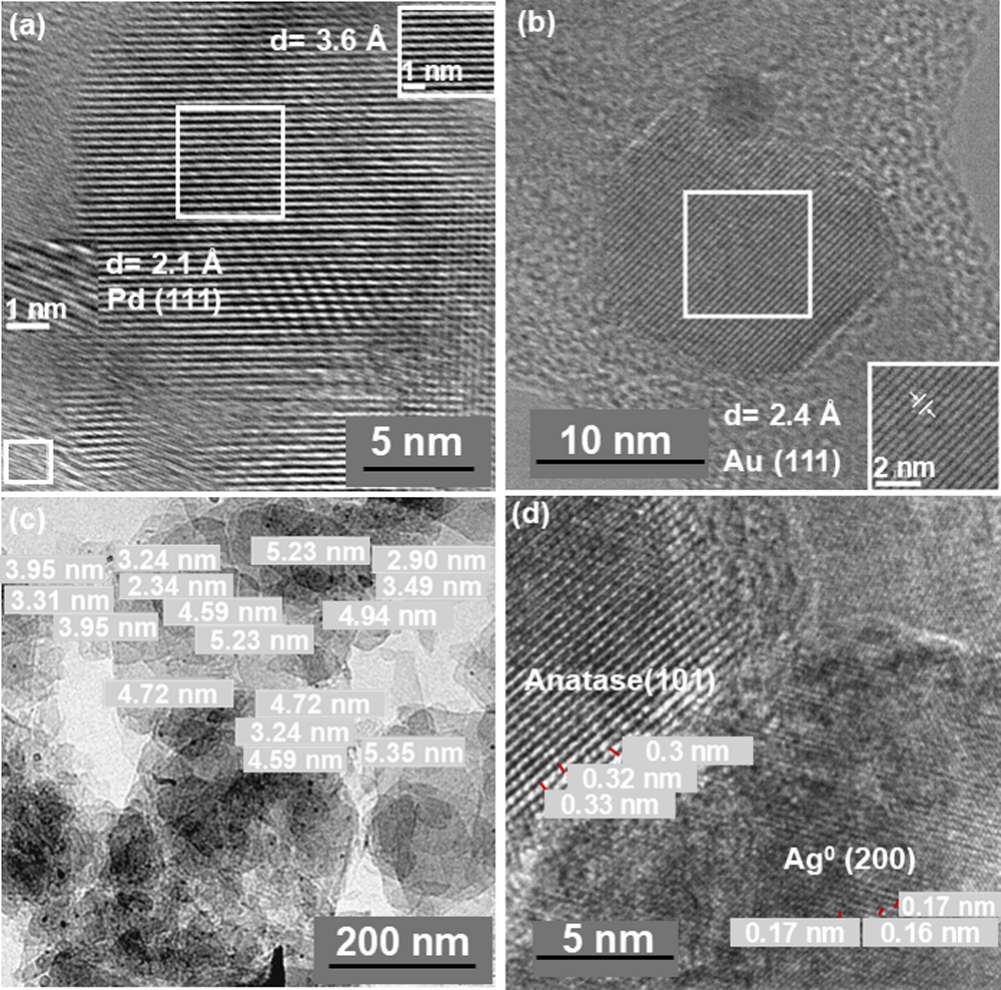
High-resolution TEM images of (a) Pd/TiO_2_, (b) Au/TiO_2_, (c) Pt/TiO_2_, and (d) Ag/TiO_2_. The insets of images (a) and (b) correspond to expansions of the regions marked by the white squares. The numbers in images (c) and (d) indicate the size of representative Pt and Ag NPs, respectively.

Figure 2 shows XRD patterns of the series of photocatalysts under study. These patterns show that TiO_2_ is constituted mainly by the anatase phase, with a minor proportion of rutile (Figure S9), and that diffraction is maintained after the metal NP deposition process. In contrast, no diffraction peaks attributable to any form of Pt and Pd were recorded in the patterns of Pd- or Pt-doped samples at 1 wt %. This is probably due to the low metal content, high dispersion, and small particle size of Pd and Pt in these samples. The absence of any characteristics Pd peak in the XRD occurred even upon increasing Pd loading to nominal percentages of 3 and 5 wt %. In contrast, in the case of Au/TiO_2_ and Ag/TiO_2_, the presence of a small peak at 2Θ values corresponding to the (111) plane of their cubic phase was observed. The presence of diffraction peaks characteristic of Au and Ag can be related to their larger particle sizes and differences in the nature of the element.

**Figure 2:**
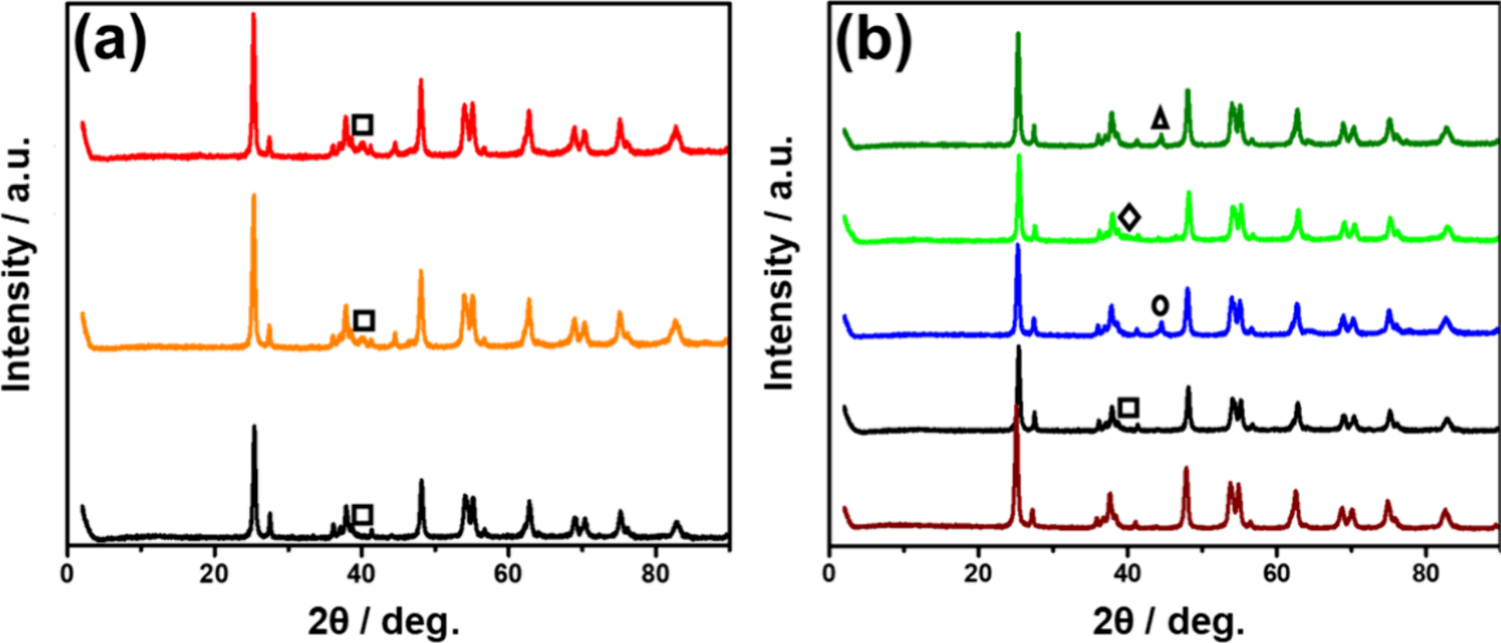
X-ray diffractograms of (a) black line 1 wt % Pd/TiO_2_, orange line 3 wt % Pd/TiO_2_, and red line 5 wt % Pd/TiO_2_. The most intense characteristic peak of Pd NPs is indicated by a black square. (b) Brown line TiO_2_, black line Pd/TiO_2_, blue line Au/TiO_2_, bright-green line Pt/TiO_2_, and dark-green line Ag/TiO_2_. The position expected for the most intense characteristic peak of each metal has been indicated on the plots: (black square)­Pd, (black circle) Au, (black diamond) Pt, and (black triangle) Ag.

Diffuse reflectance UV–vis spectroscopy is a useful tool for evaluating the light absorption and electronic characteristics of photocatalytic materials. Compared with pure P25 TiO_2_, Au/TiO_2_ exhibits a clear plasmon band in the visible region in the range from 500 to 600 nm, with an absorption maximum at 565 nm (Figure 3). The plasmon band is generated by the optical properties of gold nanoparticles due to surface plasmon resonance (SPR), which represents the movement of electrons of the conduction electrons induced by light irradiation, thanks to a considerable electric near-field. This effect could be explained by several factors such as the size and shape of the nanoparticles, the distance between them, and the dielectric properties of the surrounding medium. For this reason, Au/TiO_2_ improves the photocatalytic reaction under solar light irradiation, where UV light irradiation promotes the electrons from the valence to the conduction band in TiO_2_; then, the electrons move to the gold nanoparticles. So, the SPR effect allows an improvement of photocatalytic activity thanks to the migration of electrons, which is possible by appropriate irradiation.^55^ However, it has to be commented that the photocatalytic reactions were carried out using visible light with a 450 nm filter, and no differences due to the TiO_2_ onset shift should be expected in the present case, for which only plasmon bands should undergo photoexcitation.

**Figure 3:**
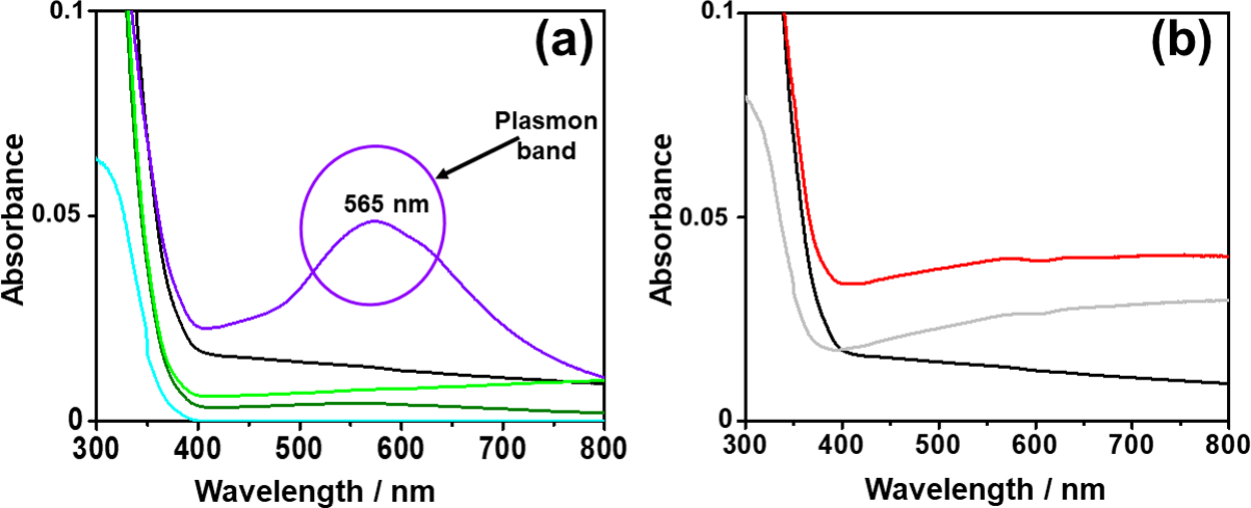
UV–vis spectra of (a) cyan line TiO_2_, black line 1 wt % Pd/TiO_2_, violet line 1 wt % Au/TiO_2_, bright-green line 1 wt % Pt/TiO_2_, and dark-green line 1 wt % Ag/TiO_2_. (b) Black line 1 wt % Pd/TiO_2_, red line 3 wt % Pd/TiO_2_, and gray line 5 wt % Pd/TiO_2_.

Without considering the plasmon bands, the absorption edge of TiO_2_ experiences a red shift upon metal deposition (see Figure 3), a fact that can be due to shallow metal doping on the TiO_2_ surface. This absorption onset shift for TiO_2_ indicates some variation of the bandgap of the metal–TiO_2_ semiconductor. However, when metals are deposited on TiO_2_ (which is the case of the present work), the electronic interactions between the metal and TiO_2_ can alter the energy levels of the valence and conduction bands. Thus, the bandgaps of the synthesized photocatalysts calculated solely from the UV–vis spectra shift might not accurately reflect the true bandgap of the metal–TiO_2_ nanoparticles, meaning that this technique is inaccurate for defective, doped, or surface-modified materials,^56^ like our case. Despite the inaccurate bandgap determination, to evaluate the trend of bandgap variation, Figure S10 reports the Tauc method plots for bare TiO_2_ and synthesized photocatalysts. Clearly, the bandgap of the metal-supported TiO_2_ photocatalysts is narrower^41^ than that of bare P25 TiO_2_, whose value was determined to be 3.3 eV. Nonetheless, the calculated bandgaps of the synthesized photocatalysts are only approximate values.

XPS analysis was carried out for the synthesized photocatalysts. The high-resolution XPS spectra of the corresponding core levels of each element for the best photocatalyst of the series (3 wt % Pd/TiO_2_) are presented in Figure 4, while for 1 wt % Au/TiO_2_, 1 wt % Pt/TiO_2_, and 1 wt % Ag/TiO_2_ photocatalysts are reported in Figures S11–S13, respectively. This figure also shows the best deconvolutions of the experimental peaks into individual components for each of the elements. Specifically, the XPS Pd 3d peak was deconvoluted into two peaks corresponding to the spin–orbit splitting of the 3d state in the Pd (3d 3/2) and Pd (3d 5/2) lines (Δ = 5.3 eV), appearing at binding energies of approximately 333.1 and 338.4 eV, respectively. The XPS Ti 2p peaks were also deconvoluted, with the peak at a binding energy of 457.2 eV attributed to Ti^4+^. No component corresponding to Ti^3+^ was found, indicating that Ti^4+^ is the only oxidation state of Ti on the surfaces of these catalysts. Regarding the O 1s spectra, a single peak centered at 528.8 eV was recorded, which is attributed to lattice oxygen (Ti–O) and chemisorbed oxygen species, such as −OH.^57^

**Figure 4:**
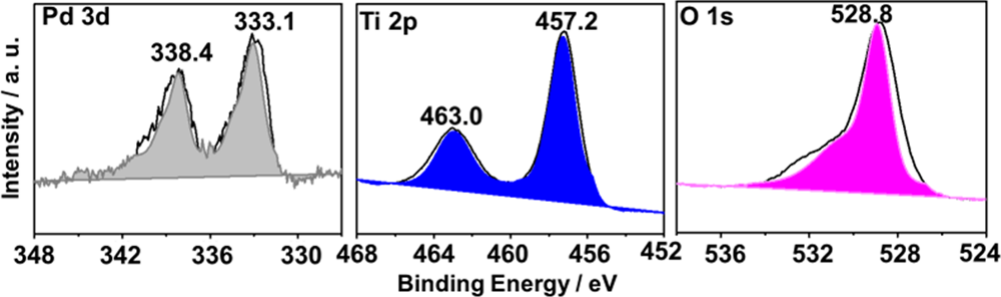
Experimental high-resolution XP spectra and the corresponding deconvolution to individual components for the best-performing photocatalyst 3 wt % Pd/TiO_2_.

### 3.2 Photocatalytic Activity

The photocatalytic tests were carried out using PS NPs (200 μg) suspended in water, together with different amounts (10, 20, 30, and 40 mg) of different photocatalysts. The total volume of the suspension was 20 mL. The photocatalysts were irradiated from the top with a visible light cutoff filter with transmission for wavelengths longer than 450 nm, using a 150 W Xe lamp through quartz for 2 h. Oxygen was removed from the test solution to create an inert atmosphere that favors reductive reactions such as hydrogen production. The presence of oxygen can act as an electron scavenger, competing with proton (H^+^) reduction and thus inhibiting efficient generation of hydrogen. By driving away external oxygen, the system minimizes side reactions and enhances the photocatalytic degradation of polystyrene, along with H_2_ production. O_2_ in the system was removed by flushing the system with argon for at least 30 min prior to irradiation. The reactor was sealed under an argon overpressure of 1 bar, and the system was magnetically stirred during the reaction.

Hydrogen generation was monitored by gas chromatography, while the size distribution of PS NPs during the photocatalytic test was monitored by dynamic laser scattering. The photocatalytic activity of the synthesized Pd/TiO_2_, Pd/CeO_2_, and Pd/C was evaluated against TiO_2_ as a benchmark. Figure 5a shows that sample Pd/TiO_2_ was the one exhibiting superior photocatalytic activity compared to the other catalysts.

**Figure 5:**
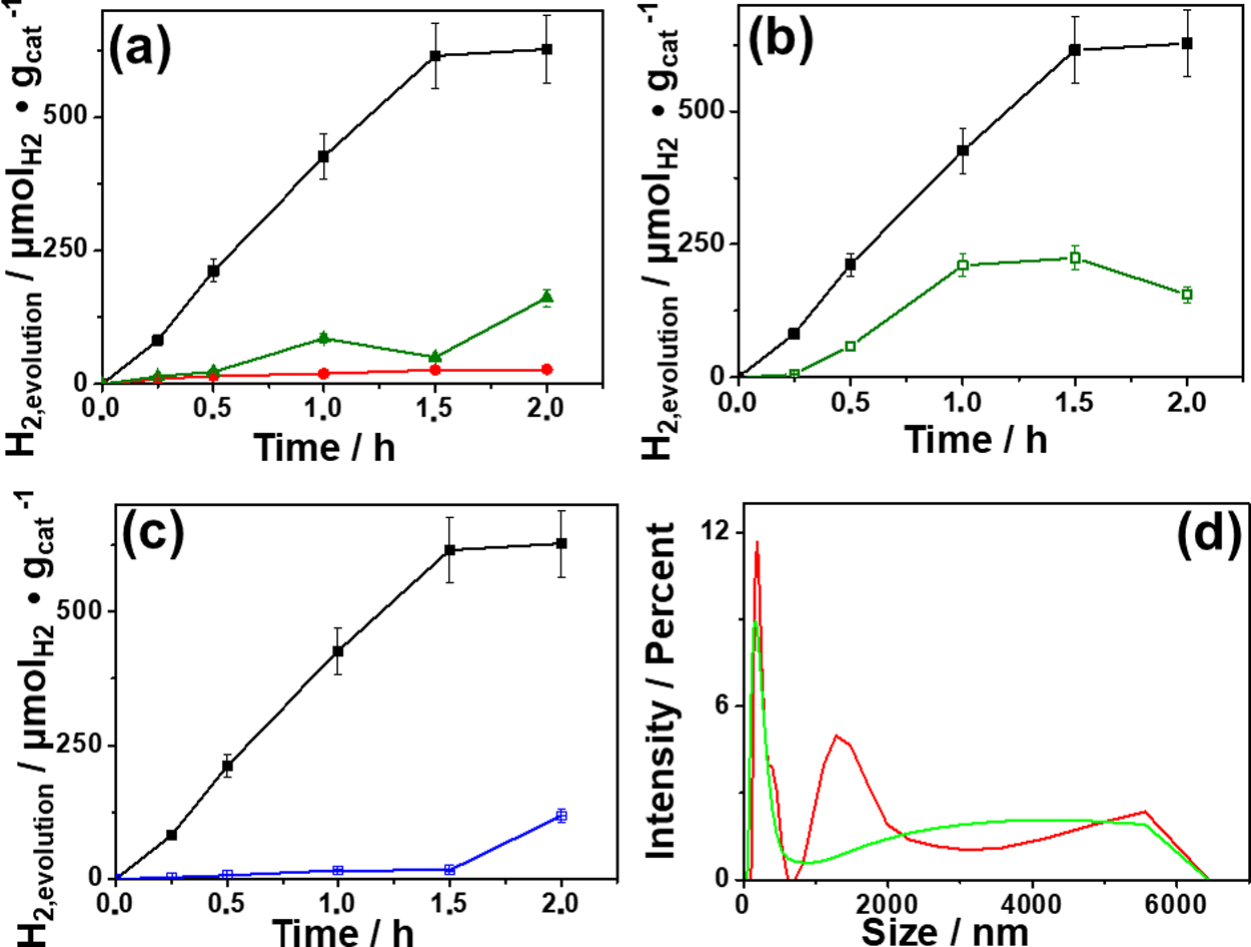
(a) Hydrogen evolution (μmol_H2_ g_cat_^–1^) vs time (h) under visible light irradiation (λ > 450 nm) from a suspension of PS NPs in H_2_O (10 mg L^–1^) using as the photocatalyst: black solid square 1 wt % Pd/TiO_2_; red solid circle 1 wt % Pd/C; or dark-green solid triangle 1 wt % Pd/CeO_2_ (1.5 mg mL^–1^). (b) Hydrogen evolution of photocatalytic tests carried out with 10 mg mL^–1^ PS NPs and 1.5 mg mL^–1^ 1 wt.% Pd/TiO_2_ as the photocatalyst, black solid square under visible light irradiation or dark-green open square without irradiation. (c) Photocatalytic tests performed with 1.5 mg mL^–1^ of 1 wt % Pd/TiO_2_ as the photocatalyst and black solid square PS NPs or blue checked box without PS NPs as sacrificial agent under visible light irradiation. (d) Size distribution measured by dynamic laser scattering of photocatalytic test at initial time red line and final time bright-green line carried out with PS NPs (10 mg L^–1^) as the sacrificial agent and 1 wt % Pd/TiO_2_ (1.5 mg mL^–1^) as the photocatalyst.

A control experiment using 1 wt % Pd/TiO_2_ in the dark was carried out and compared to the twin experiment under irradiation (Figure 5b). It was observed that stirring PS NPs in the presence of Pd/TiO_2_ leads to the removal of a notable percentage of PS NPs by adsorption on the surface of the photocatalyst, detecting a minor H_2_ evolution in the reactor head space. This detectable, minor H_2_ evolution in the dark was not observed in the absence of PS NPs, indicating that it should be due to the decomposition of a minor fraction of PS NPs catalyzed by Pd/TiO_2_. In comparison to the dark experiment, visible light irradiation of a suspension of Pd/TiO_2_ containing PS NPs produced a steady H_2_ evolution.

Furthermore, the role of PS NPs as a sacrificial electron donor was verified by comparing two identical photocatalytic tests in the absence or in the presence of PS NPs using 1 wt % Pd/TiO_2_ as the photocatalyst, under visible light irradiation for 2 h. From the results shown in Figure 5c, it can be concluded that H_2_ evolution increases significantly when PS NPs are present in comparison to pure H_2_O. This observation, together with the large PS NP adsorption percentage occurring in the dark, suggests that after adsorption on the photocatalyst surface, PS NPs undergo photoreforming, increasing the volume of H_2_ evolved. Thus, all the available data indicate that PS NPs, light, and Pd/TiO_2_ are involved in the process of H_2_ evolution.

To further investigate the role and evolution of PS NPs during the photocatalytic process under visible light irradiation, a dynamic laser scattering analysis of the particle size distribution based on diameter was performed. As shown in Figure 5d, there was a noticeable decrease in the size distribution of the NPs after 2 h irradiation in the presence of 1 wt.% Pd/TiO_2_.

To optimize the process, a series of photocatalytic irradiations were then carried out using a constant amount of PS NPs (200 μg), but varying the amount of the 1 wt % Pd/TiO_2_ photocatalyst in the range from 10 to 40 mg. Experiments were carried out using a total volume of water of 20 mL upon irradiation with visible light (λ > 450 nm) for 2 h. Figure 6 shows that 1.5 mg mL^–1^ (30 mg) of 1 wt % Pd/TiO_2_ is the optimal photocatalyst concentration for H_2_ generation. The observation of an optimal photocatalyst dose is commonly explained by the balance of two opposite effects. On one hand, increasing the concentration of the photocatalyst should increase light harvesting and the amount of photogenerated charge carriers. On the other hand, a high solid dose increases light scattering, limiting light penetration inside the photoreactor, thereby diminishing the number of photons reaching the photocatalyst, consequently diminishing the efficiency of the photocatalytic system.^58^ In our case, increasing the photocatalyst dose up to 1.5 mg mL^–1^ increases H_2_ evolution, while a higher photocatalyst amount of 2 mg mL^–1^ results in diminution in the H_2_ evolved in the system.

**Figure 6:**
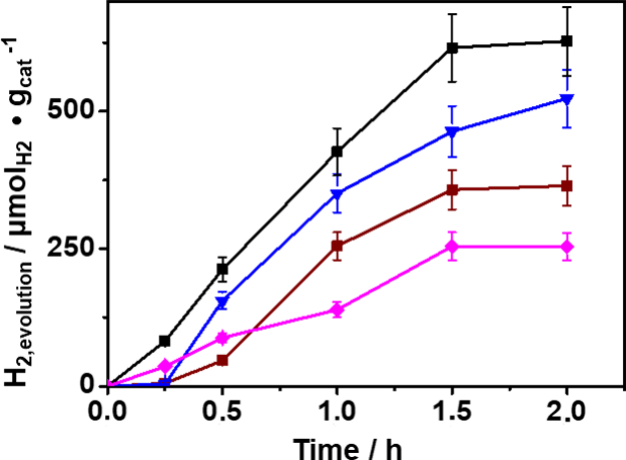
Temporal evolution of H_2_ μmol_H2_ g_cat_^–1^ upon irradiation in the presence of 1 wt % Pd/TiO_2_ as the photocatalyst with visible light irradiation (λ > 450 nm) in water containing PS NPs (10 mg L^–1^) varying photocatalyst dose: 0.5 mg mL^–1^ pink diamond; 1 mg mL^–1^ brown square; 1.5 mg mL^–1^ black square; and 2 mg mL^–1^ blue inverted triangle.

Subsequently, photocatalytic PS NP degradation tests were carried out using PS NPs (200 μg amount) and different photocatalysts at a dose of 1.5 mg mL^–1^ (Figure 7) by varying the nature of the noble metal on the TiO_2_ semiconductor. The tested samples were 1 wt % Pd/TiO_2_, 1 wt % Au/TiO_2,_ 1 wt % Pt/TiO_2_, and 1 wt % Ag/TiO_2_. Since there is some discrepancy among the experimental metal content in the photocatalysts (see Table 1), to compare their H_2_ evolution performance, a normalization per gram of metal loaded on the photocatalyst surface instead of a simple gram of overall nanoparticle photocatalyst has been done. This criterion has been applied only in this case to compare the performance of the different loaded metals, but in the other cases, where only Pd has been loaded, the overall weight of Pd/TiO_2_ nanoparticles has been used as the reference. It was found that among the four loaded metals, Pd/TiO_2_ was the best-performing photocatalyst, promoting higher H_2_ generation and a deeper transformation of PS NPs. The enhanced photocatalytic activity of Pd/TiO_2_ can be attributed to the role of Pd metal as an electron buffer, which reduces the recombination of electron–hole pairs, and as a catalyst promoting H_2_ gas evolution. An additional reason contributing to the higher efficiency of Pd/TiO_2_ nanoparticles is the narrowing of the optical bandgap (Figure S10) resulting from Pd doping of TiO_2_ during the deposition of the Pd NPs. This narrower band gap may allow more hot electrons to be received from Pd NPs into the TiO_2_ conduction band.

**Figure 7:**
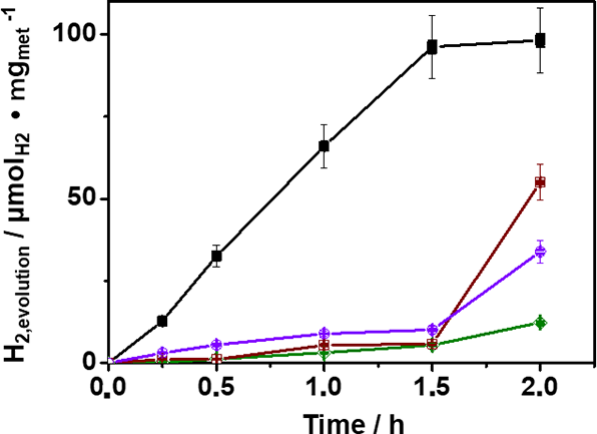
Comparison of temporal H_2_ evolution (μmol_H2_ mg_met_^–1^) of the different metals loaded on TiO_2_ nanoparticles (visible light irradiation (λ > 450 nm), PS NPs 10 mg L^–1^ suspended in water (20 mL) and photocatalyst 1.5 mg mL^–1^ (30 mg) 1 wt % Pd/TiO_2_ black solid square, 1 wt % Au/TiO_2_ violet open circle, 1 wt % Pt/TiO_2_ green open diamond, and 1 wt % Ag/TiO_2_ brown open square).

Photocatalytic tests of PS NP (200 μg) degradation in H_2_O (20 mL) were then carried out using Pd/TiO_2_ photocatalysts having different Pd loadings on the TiO_2_ support. Specifically, a series of Pd/TiO_2_ photocatalysts named 1, 3, and 5 wt % Pd/TiO_2_ were prepared. Figure 8a shows that the 3 wt % Pd/TiO_2_ sample exhibits a marked improvement in activity, considering both H_2_ generation and diminution of the average size of PS NPs. Furthermore, the decrease in the average particle size (based on diameter) determined by dynamic laser scattering Figure 8b was additionally supported by measurements of the NP size distribution in FE-SEM images (Figure 8d–g). The superior performance of the 3 wt % Pd/TiO_2_ has to be due to a balance among various factors, including a positive enhancement of charge separation efficiency and H_2_ gas evolution due to larger Pd amount.

**Figure 8:**
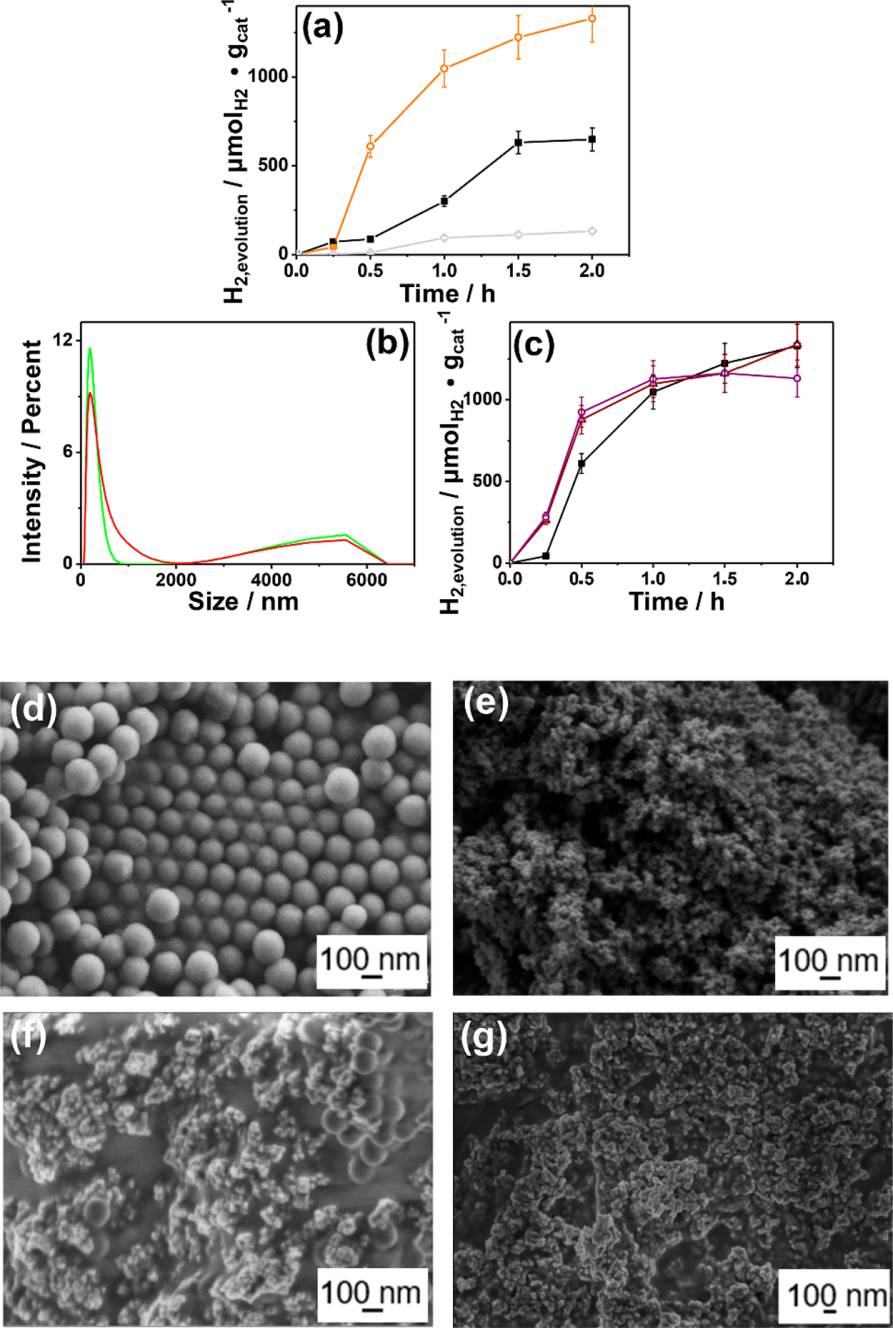
(a) Diameter size distribution analyzed by dynamic laser scattering of PS NPs in water before (green line) and after 2 h irradiation with visible light using 3 wt.% Pd/TiO_2_ (1.5 mg mL^–1^) as the photocatalyst (red line). (b) Plot of H_2_ evolution μmol_H2_ g_cat_^–1^ in the time under visible light (λ > 450 nm) irradiation of an aqueous suspension of PS NPs (10 mg L^–1^) in the presence of 1 wt % Pd/TiO_2_ black solid square, 3 wt % Pd/TiO_2_ orange open circle, or 5 wt % Pd/TiO_2_ gray open diamond (1.5 mg mL^–1^) as the photocatalyst. (c) Photocatalytic H_2_ evolution (μmol_H2_ g_cat_^–1^) for three consecutive uses (the photocatalyst is washed and dried at 200 °C overnight before use) of 3 wt % Pd/TiO_2_ (first time black solid square, second time brown open triangle, and third time violet open circle). FE-SEM images of (d) PS NPs, (e) fresh 3 wt % Pd/TiO_2_, (f) fresh 3 wt % Pd/TiO_2_ (1.5 mg mL^–1^) mixed with PS NPs (10 mg mL^–1^) without irradiation and (g) sample after visible light irradiation.

Regarding stability, the same 3 wt % Pd/TiO_2_ sample was subjected to three consecutive uses as a photocatalyst under visible light irradiation (λ > 450 nm), with the temporal profile of hydrogen evolution being monitored (Figure 8c). A slight decrease in the final reaction rate and overall hydrogen production was observed, which could be attributed to an experimental analytical error or minor deactivation of the photocatalyst after three consecutive uses. Moreover, elemental ICP analysis of the aqueous phase showed the absence of Pd in the liquid in detectable amounts, indicating that there was no Pd leaching from the solid to the liquid phase during the photocatalytic reactions.

XPS analyses of the 3 wt % Pd/TiO_2_ sample after its use as a photocatalyst showed changes in the binding energy values of Pd 3d, Ti 2p, and O 1s peaks in the process. Figure 9 presents these changes. Particularly informative is the increase in the binding energy of the Pd 3d core level, which is compatible with the occurrence of the partial oxidation of Pd atoms in the process. This would indicate the accumulation of holes and removal of hot electrons during the process, probably by injecting them into the TiO_2_ conduction band. In the case of O 1s, the decrease in the binding energy value could be related to the presence of weakly adsorbed H_2_O and other oxygenated species on the surface.

**Figure 9:**
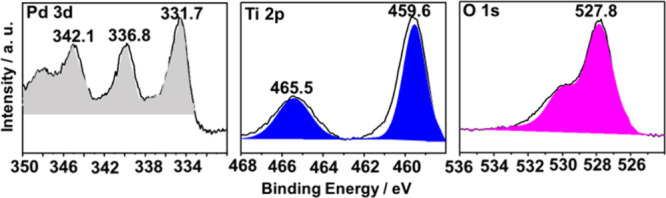
Experimental high-resolution XP spectra and the corresponding deconvolution to individual components for the best-performing photocatalyst 3 wt % Pd/TiO_2_ after 2 h of after visible light irradiation.

Worth noting is the evolution of PS NPs upon contacting with fresh 3 wt % Pd/TiO_2_ and after 2 h irradiation. This evolution can be followed by FE-SEM images. As shown in Figure 8d, PS NPs are ideal spheres of about 150 nm, in agreement with the specifications of the commercial sample. Upon contacting with a 6.6-fold lower mass of the 3 wt % Pd/TiO_2_ photocatalyst, FE-SEM images in Figure 8f reveal a strong association between the two components: PS NPs and the photocatalyst. Inspection of these FE-SEM images before irradiation can still identify spheres corresponding to PS NPs. In contrast, upon 2 h of irradiation, the FE-SEM images show that the spheres present in PS NPs have almost completely disappeared, and the morphology of the solid is more similar to that of pristine 3 wt %Pd/TiO_2_, as revealed from the comparison between Figure 8e,g. This clear change in morphology is direct visual evidence of the chemical transformation undergone by PS NPs during the course of the irradiation, while H_2_ is evolving from the system. In addition, micro-GC analysis revealed the formation of CO_2_ and CH_4_, further confirming the photocatalytic degradation of PS NPs.

In order to rank the photocatalytic activity of 3 wt % Pd/TiO_2_ in the current state of the art, Table 2 provides a summary of the performance of other reported photocatalysts for H_2_ generation from microplastics.

**Table 2:** Summary of the Photocatalytic H-Evolution Data upon Irradiation of Aqueous Suspensions of Microplastics

photocatalysts	polymer	light	photoreforming experiment	H_2_ rate	references
SiC-1%g-C_3_N_4_ composites	PET	simulated solar light (Osram Ultra Vitalux 300 W, irradiance of 10.7 mW cm^–2^)	50 mg of photocatalyst, 40 mL deionized water, 10 mL of a solution containing 50 mg of PET solubilized in 10 M NaOH. Irradiation time 5 h	18 μmolH_2_ g_cat_^–1^ h^–1^	Iapichino et al.^59^
TiO_2_/Au, 5 wt.% 3 M KOH at 25 °C TiO_2_/Au, 5 wt % 10 M KOH at 40 °C TiO_2_/Pt, 5 wt % 3 M KOH at 25 °C	PET	high-pressure UV mercury vapor lamp (60 mW cm^–2^)	0.5 g photocatalyst, 50 mL of deionized water, 0.5 g of pretreated polymer in 3 M KOH and 50 mL quartz round-bottom flask. Irradiation time 20 h	22.13 ± 1.78, 55.41 ± 1.39, and 25.62 ± 0.39 μmolH_2_ g_cat_^–1^, respectively	Edirisooriya et al.^33^
Au/TiO_2_, 5 wt % 3 M KOH at 25 °C	PET	visible light from a metal halide lamp (45 mW cm^–2^)	0.5 g photocatalyst, 50 mL of deionized water, 0.5 g of pretreated polymer in 3 M KOH, a 50 mL quartz round-bottom flask. Irradiation time 20 h	13.74 ± 0.69 μmolH_2_ g_cat_^–1^	Edirisooriya et al.^33^
Pt/TiO_2_ and EtOH	PET LDPE PS	UV Hg lamp (160 W PUV-10) a dominant peak at 365 nm	10 mL of pretreated polymer (3:2 ethanol: water with 5%(w/w) NaOH added to 0.50 g of polymer), 0.5 g of the catalyst, irradiation time 48 h.	1.93 × 10^3^, 3.09 × 10^3^ and 2.10 × 10^2^ μmol_H2_ g_cat_^–1^, respectively.	Edirisooriya et al.^60^
RP@CoxPy/Cd_0.5_Zn_0.5_S	PLA PET	visible light by (300 W Xe lamp) with a cutoff filter (λ > 420 nm)	200 mg of plastic suspended in 25 mL of a 10 M NaOH aqueous solution, 25 mg of photocatalyst. Irradiation time 5 h at 35 °C.	503.9 and 74.4 μmol h^–1^, respectively	Li et al.^61^
3 wt % Pd/TiO_2_	PS	visible light by (150 W Xe lamp, irradiation of 100 mW cm^–2^) with a cutoff filter (λ > 450 nm)	200 μg PS NPs suspended in deionized water with 30 mg of photocatalyst. Irradiation time 2 h at 25 °C.	1329.76 μmolH_2_ g_cat_^–1^	this article

Although comparisons with reported data have to be always taken cautiously, due to the variety of experimental conditions employed in each case, it is important to underline that different types of nanoplastics can exhibit different photodegradation and conversion efficiencies. In addition, there are different parameters to be considered, such as temperature, pH, reactor design, solvent effects, and light intensity, which can influence the nanoplastics photoreforming process.^62^ However, the data in Table 2 show that 3 wt % Pd/TiO_2_ is among the best-performing materials, approaching the photocatalytic activity of the Pt/TiO_2_ used also for PS polymer, but with the remarkable difference that, in the present case, the irradiation is carried out in the visible region (light of wavelength longer than 450 nm, while in that case, a UV lamp is used) and without submitting the plastic suspension to any pretreatment. Thus, it can be concluded that the present 3 wt % Pd/TiO_2_ system is far more advantageous compared to the reported photocatalysts.

### 3.3 Photoreaction Mechanism

It has been reported that the photocatalytic degradation of PS NPs by TiO_2_ follows a complex mechanism, involving several elementary steps.^24^ A similar mechanism, adapted to the presence of Pd NPs on TiO_2_, is proposed to occur in the present case, as shown in the simplified scheme in Figure 10, also based on the mechanism proposed by several authors.^23,24,30^ Thus, when exposed to visible light, the Pd nanoparticles should enhance the absorption of near-edge photons by TiO_2_, facilitating the generation of electron–hole pairs by photons in the 400–500 nm range (eq 1).

$$\mathrm{Semiconductor}+h\nu \to \mathrm{semiconductor}\times (e^{-}+h^{+})$$ 1

**Figure 10:**
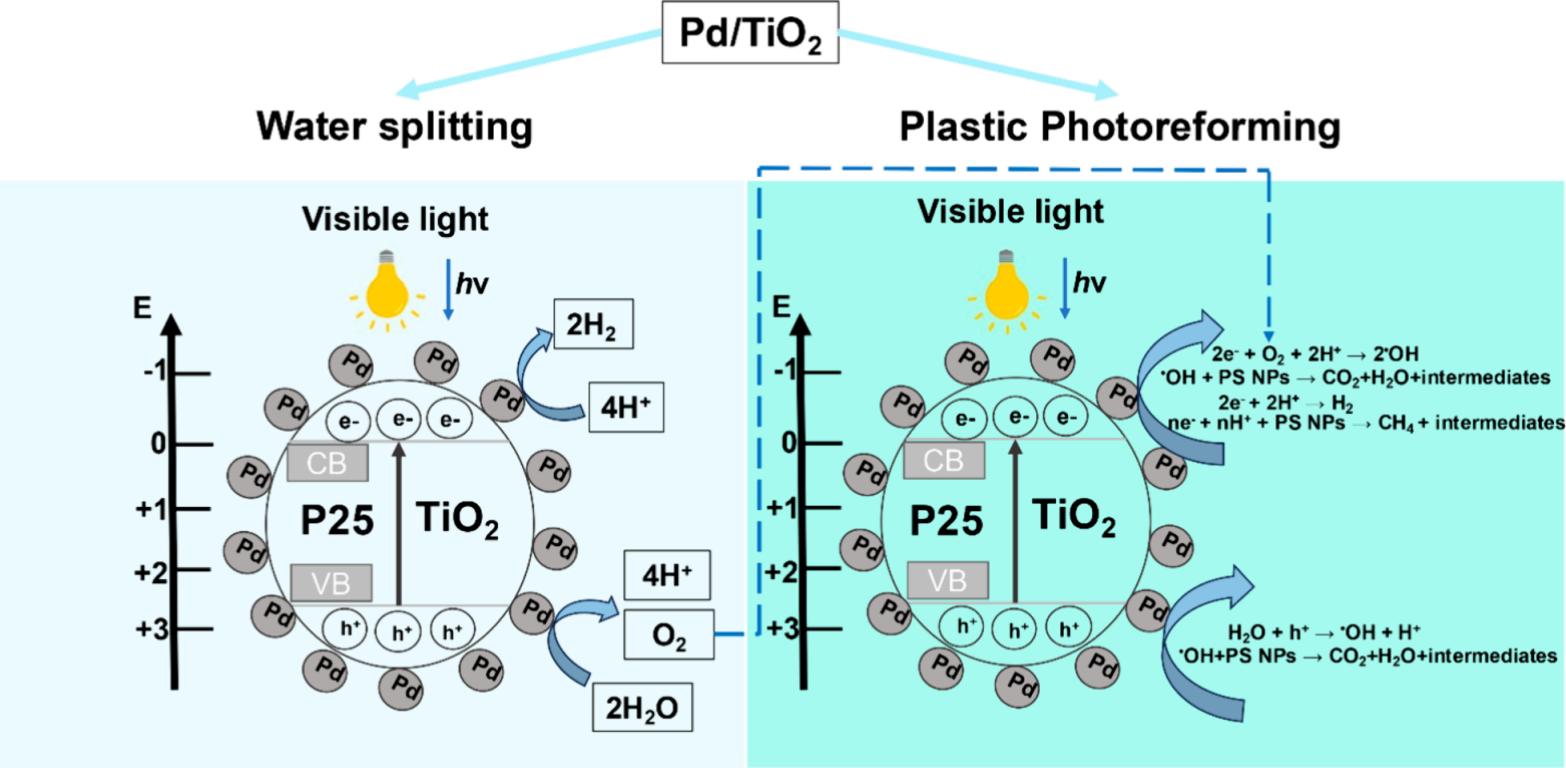
Proposed mechanism for the photocatalytic H_2_ evolution from aqueous dispersion of PS NPs (detailed mechanism explained in the text). The energy levels (E) are in volts versus the normal hydrogen electrode (NHE).

On the other hand, photon absorption by the Pd plasmon band extending in the visible-near IR region will produce hot electrons, some of which could be injected into the TiO_2_ conduction band.

These electrons and holes formed will be responsible for parallel and series reactions on the nanoparticle photocatalyst, resulting in hydrogen evolution by electrons and nanoplastics photoreforming, with consequent H_2_ evolution and formation of dehydrogenated intermediates. Photogenerated holes, either at Pd NPs or TiO_2_ valence bands, will then be responsible for PS NP oxidation. Photocatalytic hydrogen evolution and nanoplastics photoreforming and other possible reactions of holes and electrons are indicated in the following equations, where the PS NP photoreforming is reported in eqs 8–10 according to Zhang et al.^63^ and Anh Nguyen et al.^30^

Hydrogen evolution:

$$2e^{-}+2H_{2}O\to H_{2}+2{\mathrm{OH}}^{-}$$ 2

Water oxidation:

$$H_{2}O+h^{+}\to \cdot \mathrm{OH}+H^{+}$$ 3

$$4h^{+}+2H_{2}O\to O_{2}+4H^{+}$$ 4

Generation of reactive oxygen species:

$$e^{-}+O_{2}\to \cdot O_{2}^{-}$$ 5

$$\cdot {O_{2}}^{-}+e^{-}+2H^{+}\to H_{2}O_{2}$$ 6

$$H_{2}O_{2}+h\nu \to 2\cdot \mathrm{OH}$$ 7

PS NP photoreforming:

$$h^{+}+\mathrm{PS}\;\mathrm{NPs}\to [\mathrm{PS}\;\mathrm{NPs}{]}^{\cdot +}\to H\cdot +[\mathrm{PS}\;\mathrm{NPs}{]}^{+}\to \mathrm{intermediates}\;\mathrm{with}\;\mathrm{less}\;\mathrm{hydrogen}\;\mathrm{content}$$ 8

PS NP photooxidation:

$$\cdot \mathrm{OH}+\mathrm{PS}\;\mathrm{NPs}\to {\mathrm{CO}}_{2}+H_{2}O+\mathrm{intermediates}\;\mathrm{with}\;\mathrm{less}\;\mathrm{carbon}\;\mathrm{atoms}$$ 9

PS NP photoreduction:

$$ne^{-}+nH^{+}+\mathrm{PS}\;\mathrm{NPs}\to {\mathrm{CH}}_{4}+\;\mathrm{intermediates}\;\mathrm{with}\;\mathrm{less}\;\mathrm{carbon}\;\mathrm{atoms}$$ 10

H_2_ evolution from parallel water splitting and photoreforming:

$$2e^{-}+2H^{+}\to H_{2}$$ 11

To evaluate the role of ROS (reactive oxygen species) in the photodegradation of plastic, Lin et al.^64^ conducted electron paramagnetic resonance (EPR) and chemical quenching experiments that confirmed the significant contribution of ·OH on plastic degradation, as previously illustrated in the reaction mechanism discussed above.

Additional evidence supporting the role of the photocatalyst in the reaction pathway is provided by the accumulation of holes on Pd, which causes partial oxidation of this metal, as observed by XPS analysis of the used Pd/TiO_2_ photocatalyst. In this mechanism, with the photoinduced generation of electrons and holes, PS NPs will be acting as sacrificial electron donors, quenching photogenerated holes (eq 8). Although the photocatalytic H_2_ generation in pure water is very low (Figure 5c), its occurrence confirms that water oxidation by holes (eqs 4 and 5) will generate hydroxyl radicals as primary reactive oxygen species. In addition, ROS can also be derived from O_2_ reduction (eqs 5–8). ROS can attack PS chains, particularly at benzylic positions, promoting the degradation of the PS NPs. In this way, the photocatalytic process produces plastic degradation by photogenerated holes and electrons as well as ROS, compatible with the changes in PS NP morphology shown in Figure 8g, while simultaneously providing H_2_ by H^+^ reduction.

The interaction between the photocatalyst and PS NPs was supported by FE-SEM images shown in Figure 8f and by measuring the zeta potential of both particles in the H_2_O suspension at neutral pH. As shown in Figure 11a, both the individual PS NPs and the photocatalysts exhibit high zeta potential values, indicating good particle dispersion. In contrast, the combination of PS NPs and the photocatalyst shows a zeta potential value close to zero, which suggests a significant neutralization by association between them,^65^ which is confirmed by FE-SEM images (Figures 8f and 11b). The interaction between PS NPs and the photocatalyst can be explained by the loose polymeric structure and the presence of π–π interactions in the PS NPs,^66^ as well as by the reduced size, which enhances various van der Waals interactions with the surrounding environment and modifies the electronic structure of the material.^67^

**Figure 11:**
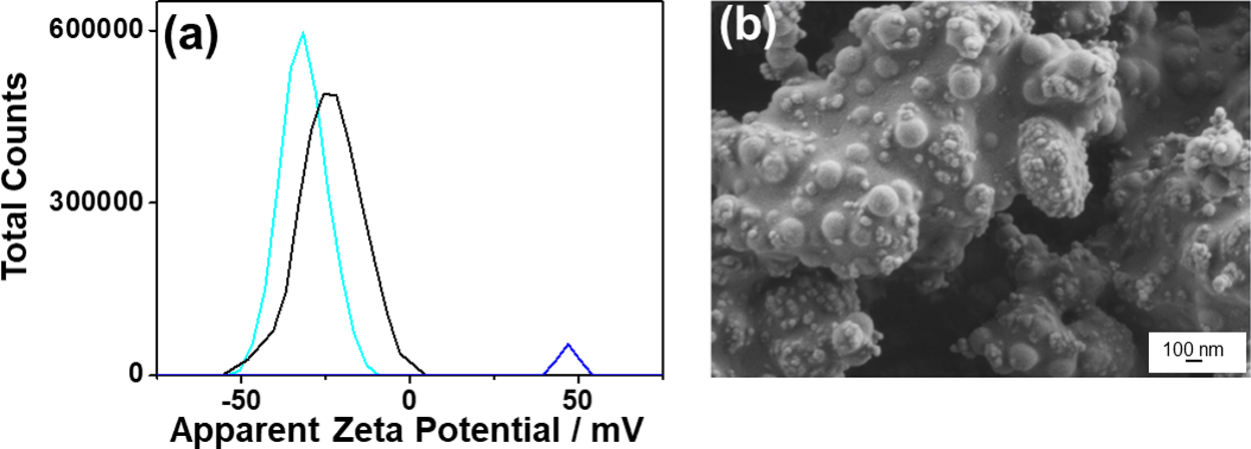
(a) Zeta potential analysis at neutral pH of the 3 wt % Pd/TiO_2_ photocatalyst black line, PS NPs cyan line, and the combination of 3 wt % Pd/TiO_2_ with PS NPs blue line. (b) FE-SEM image of 3 wt % Pd/TiO_2_ (1.5 mg mL^–1^) being exposed to PS NPs (10 mg mL^–1^) at initial time of visible light irradiation.

### 3.4 Economic Evaluation of Nanoplastics Photoreforming

The photoreforming of nanoplastics is a possible solution for the simultaneous generation of renewable energy and the photodegradation of a recalcitrant pollutant.^68,69^ To evaluate the nanoplastic photoreforming process at an industrial from lab-scale level, including both water splitting and nanoplastics reforming (as reported in the hypothesized reaction mechanism in paragraph Section 3.3), Gunawan et al.^70^ employed the solar-to-hydrogen (STH) parameter, which is commonly accepted to evaluate the efficiency of hydrogen production performance. The STH efficiency (eq 12) is defined as:

$$\mathrm{STH}=\frac{R_{H_{2}}\times |\Delta G_{\mathrm{use}}|}{P\times S}\times 100\%$$ 12

where *R*_H2_ is the hydrogen evolution rate (mol s^–1^), *P* is the light intensity (W m^–2^), *S* is the irradiation area (m^2^), and Δ*G*_use_ is the Gibbs free energy of hydrogen combustion ≈ −237 kJ mol^–1^ under standard conditions.^70^

Considering this parameter, the STH efficiency for industrial applications should be in the range 15–25% or more, while currently, it is reported well below 10%.^62^ In our case, a value of 0.6% was estimated using the best-performing conditions. It is too small efficiency, being that the experimental system of a very small laboratory scale (reactor volume 51 mL) is used only as basic research. However, to improve STH efficiency, a good strategy could be improving visible-light absorption, among the three noble metals as Pd, Au, and Pt nanoparticles employed in this study. As shown in Figure 3a, Pd has the highest light absorption after Au in the visible range, so it is expected, it could be the metal of interest for H_2_ generation from PS NPs since the comparison of current prices shows that Pd has the lowest price (about 29 euros per gram against 90 for Au and 35 for Pt). This could be a good starting point in the choice of the doped nanoparticle photocatalyst for eventual industrial-scale applications.

## 4 Conclusions

The use of photocatalytic nanoparticles has been shown to be a potentially viable strategy for treating nanoplastics in wastewater while evolving H_2_ from photoreforming. Various metal nanoparticles (Pd, Au, Pt, and Ag) were deposited on TiO_2_ nanoparticles, and the characterization by TEM and ICP analyses confirmed the deposition of metal NPs on TiO_2_, which was also reflected by color changes. No significant changes in the TiO_2_ crystallinity occurred during the metal NP deposition, as verified by XRD analysis. The failure to detect diffraction peaks corresponding to the metals at 1 wt % loading was probably due to the low metal concentration and the small particle size of the resulting metal NPs. The surface chemical states of the 3 wt % Pd/TiO_2_ photocatalyst were analyzed by XPS, which showed the presence of Pd^0^ and Ti^4+^ in the nanomaterial.

The 3 wt.% Pd/TiO_2_ photocatalyst exhibited the highest activity for hydrogen generation under visible light irradiation, achieving a production of 1329.76 μmol_H2_ gcat^–1^ and a reduction in the average PS NP diameter. These data compare favorably with existing literature and derive from the strong association between Pd/TiO_2_ and PS NPs, as observed by scanning electron microscopy. The 3 wt % Pd/TiO_2_ nanomaterial was reused three times without decay in its photocatalytic H_2_ evolution activity, while observing a certain Pd oxidation by XPS analysis, which could be explained by an accumulation of holes and the removal of hot electrons during the process, probably by injecting them into the TiO_2_ conduction band.

In addition, a photocatalytic reaction mechanism was hypothesized, which included a series of parallel and series reactions on the nanoparticle photocatalyst, resulting in hydrogen evolution by electrons and nanoplastics photoreforming with consequent H_2_ evolution and the formation of dehydrogenated intermediates. Photogenerated holes, either at Pd NPs or TiO_2_ valence bands, as well as the ·OH radical, will be then responsible for PS NP oxidation.

Overall, the 3 wt % Pd/TiO_2_ photocatalyst has been proven to be an effective nanomaterial for hydrogen generation using polystyrene nanoplastic waste, which acted as a sacrificial agent.

## Supplementary Material



## Notes

STEM images and particles counting metal size of Pd/TiO_2_ nanoparticles; high-resolution TEM images and particle counting metal size of Au/TiO_2_, Pt/TiO_2_, and Ag/TiO_2_ nanoparticles; SEM-EDX analysis of Pd/TiO_2_ nanoparticles; STEM-EDX analysis of Au/TiO_2_, Pt/TiO_2_, and Ag/TiO_2_ nanoparticles; XRD pattern of P25 TiO_2_ nanoparticles obtained experimentally and compared with standard PDF card references; Tauc plots for bandgap energy (*E*_g_) determination of bare TiO_2_ and approximate bandgaps of metal-TiO_2_ photocatalysts; experimental high-resolution XP spectra; and corresponding deconvolution to individual components for 1 wt % Au/TiO_2_, Pt/TiO_2_, and Ag/TiO_2_ (PDF)
