# Supplementary material for: Visible-Light-Driven Photocatalytic Hydrogen Production from Polystyrene Nanoplastics Using Pd/TiO2 Nanoparticles
Source: ACS Appl Nano Mater. 2025 Jul 16;8(29):14720–32. doi: 10.1021/acsanm.5c02376 (PMC12308734; doi:10.1021/acsanm.5c02376)
Supplement: Supplementary file 1 [file an5c02376_si_001.pdf]

Supporting Information

Visible-Light-Driven Photocatalytic Hydrogen Production from Polystyrene Nanoplastics Using Pd/TiO_2 Nanoparticles

Angela Severino[b,], Abdessamad Grirrane[a], María Cabrero-Antonino[a], Cristina Lavorato[b], Pietro Argurio[b], Raffaele Molinari[b], Hermenegildo García[a,*]*

[a] Instituto Universitario de Tecnología Química, Universitat Politecnica de Valencia-Consejo Superior de Investigaciones Científicas, Av. De los Naranjos s/n, 46022 Valencia (Spain).

[b] Department of Environmental Engineering (DIAm), University of Calabria, via P. Bucci, Cubo 44/A, 87036 Rende (CS), Italy.

Corresponding authors: hgarcia@qim.upv.es, angela.severino@unical.it

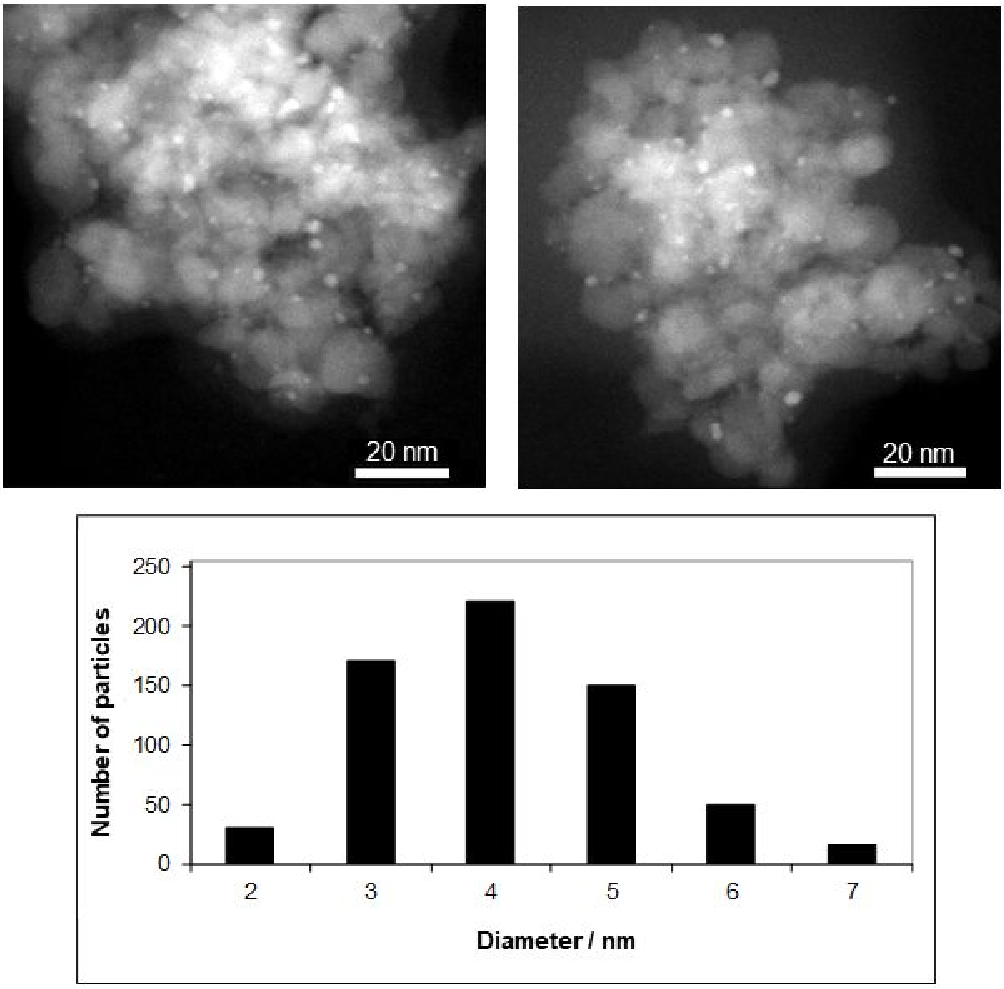

Figure S1. STEM images and particles counting metal size of Pd/TiO_2 nanoparticles.

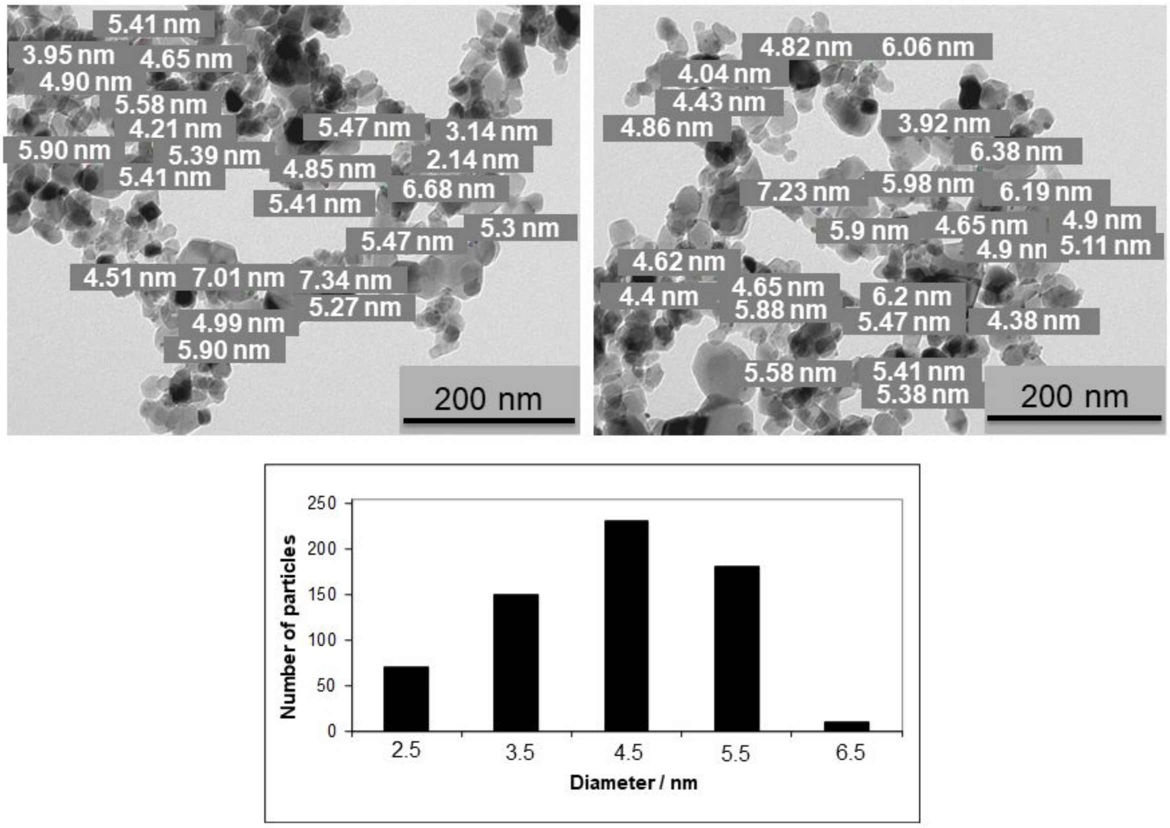

Figure S2. High Resolution TEM images and particle counting metal size of Au/TiO_2 nanoparticles.

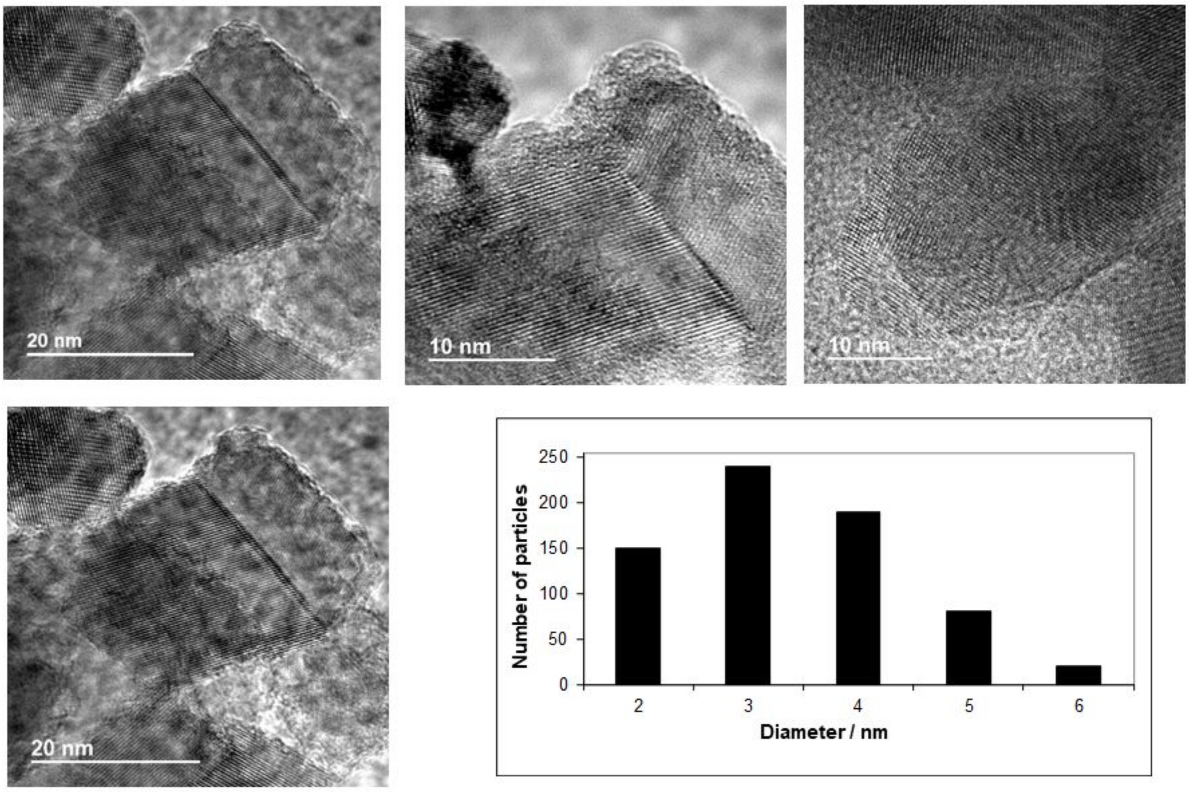

Figure S3. High Resolution TEM images and particle counting metal size of Pt/TiO_2 nanoparticles.

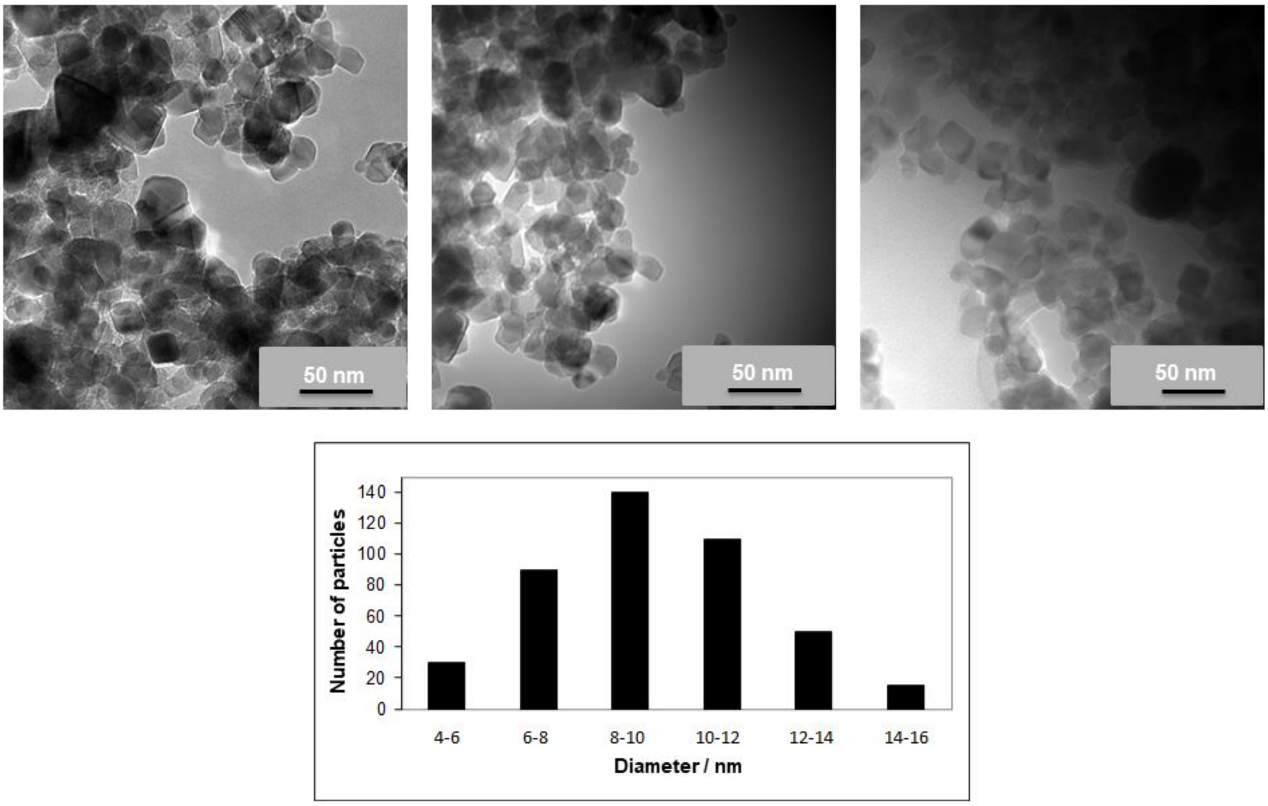

Figure S4. High Resolution TEM images and particle counting metal size of Ag/TiO_2 nanoparticles.

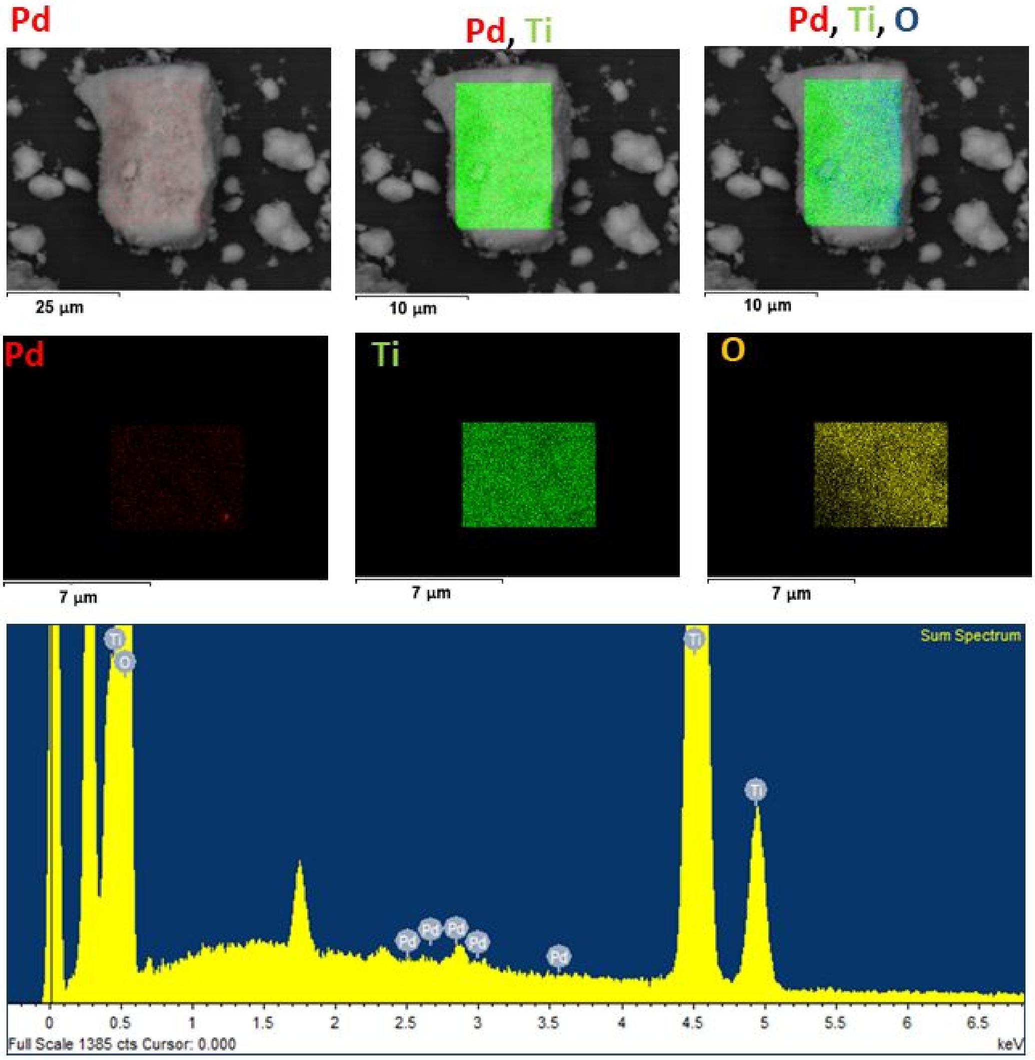

Figure S5. SEM - EDX analysis of Pd/TiO_2 nanoparticles.

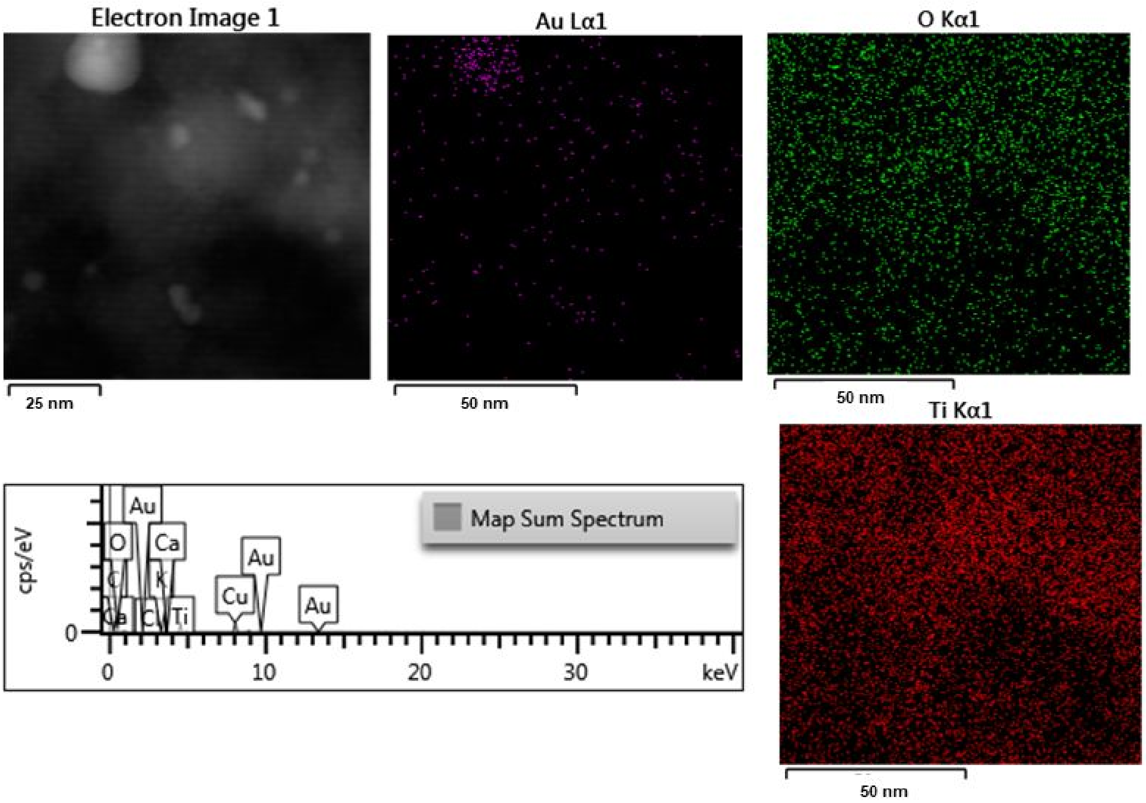

Figure S6. STEM - EDX analysis of Au/TiO_2 nanoparticles.

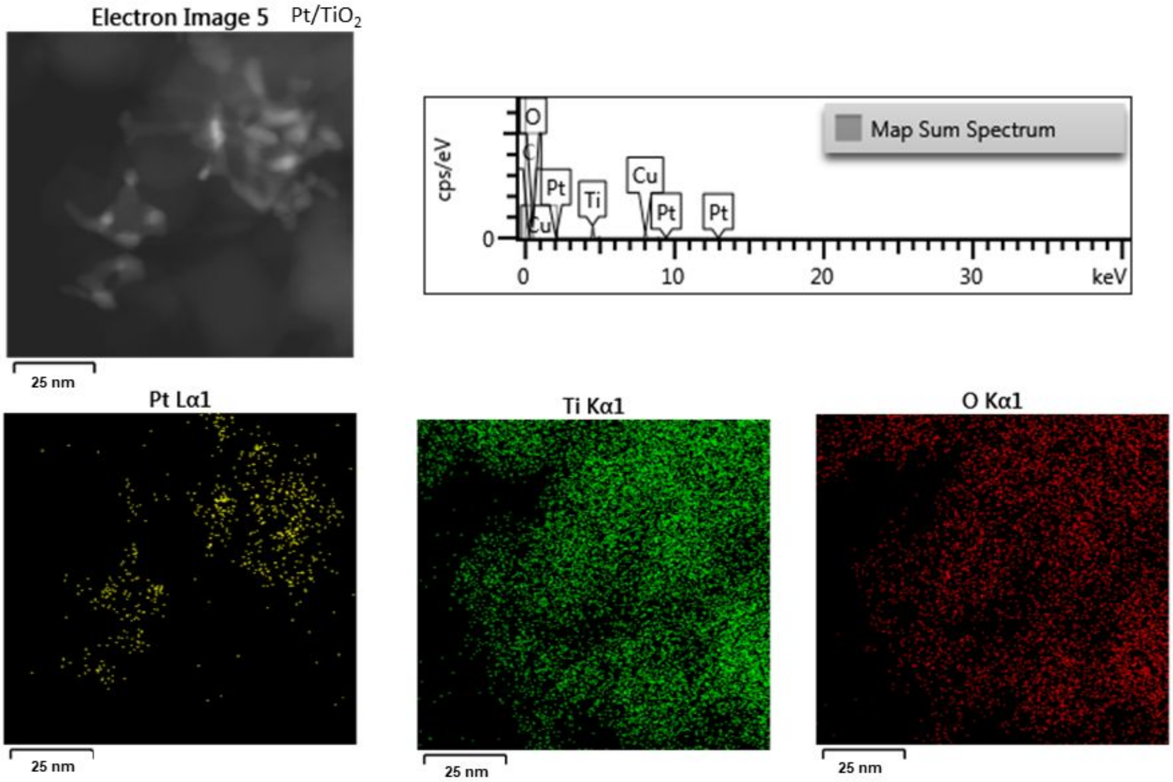

Figure S7. STEM - EDX analysis of Pt/TiO_2 nanoparticles.

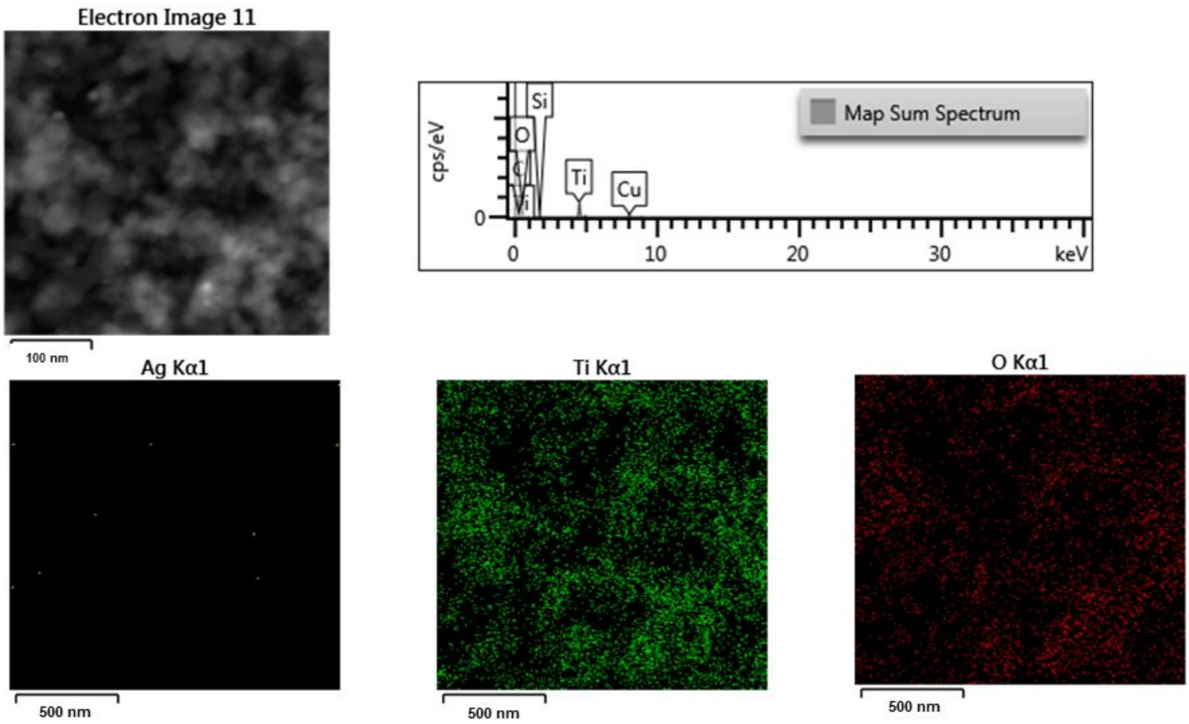

Figure S8. STEM - EDX analysis of Ag/TiO_2 nanoparticles.

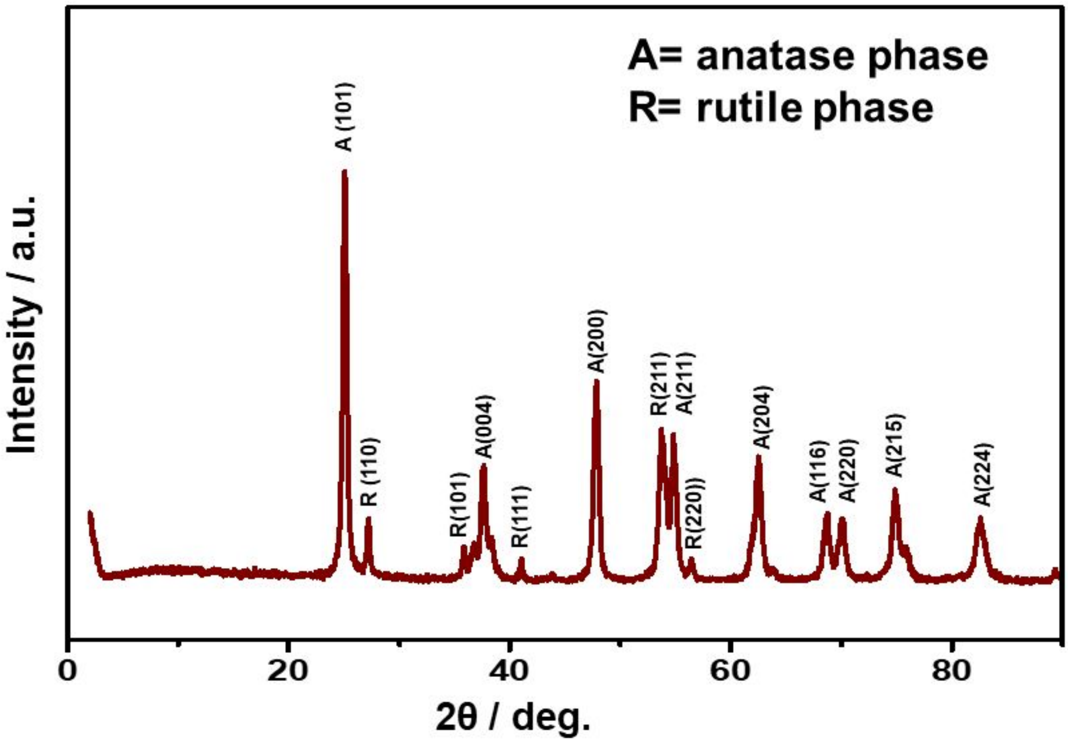

Figure S9. XRD pattern of commercially available P25 TiO_2 nanoparticles experimentally obtained, compared with standard PDF card references.

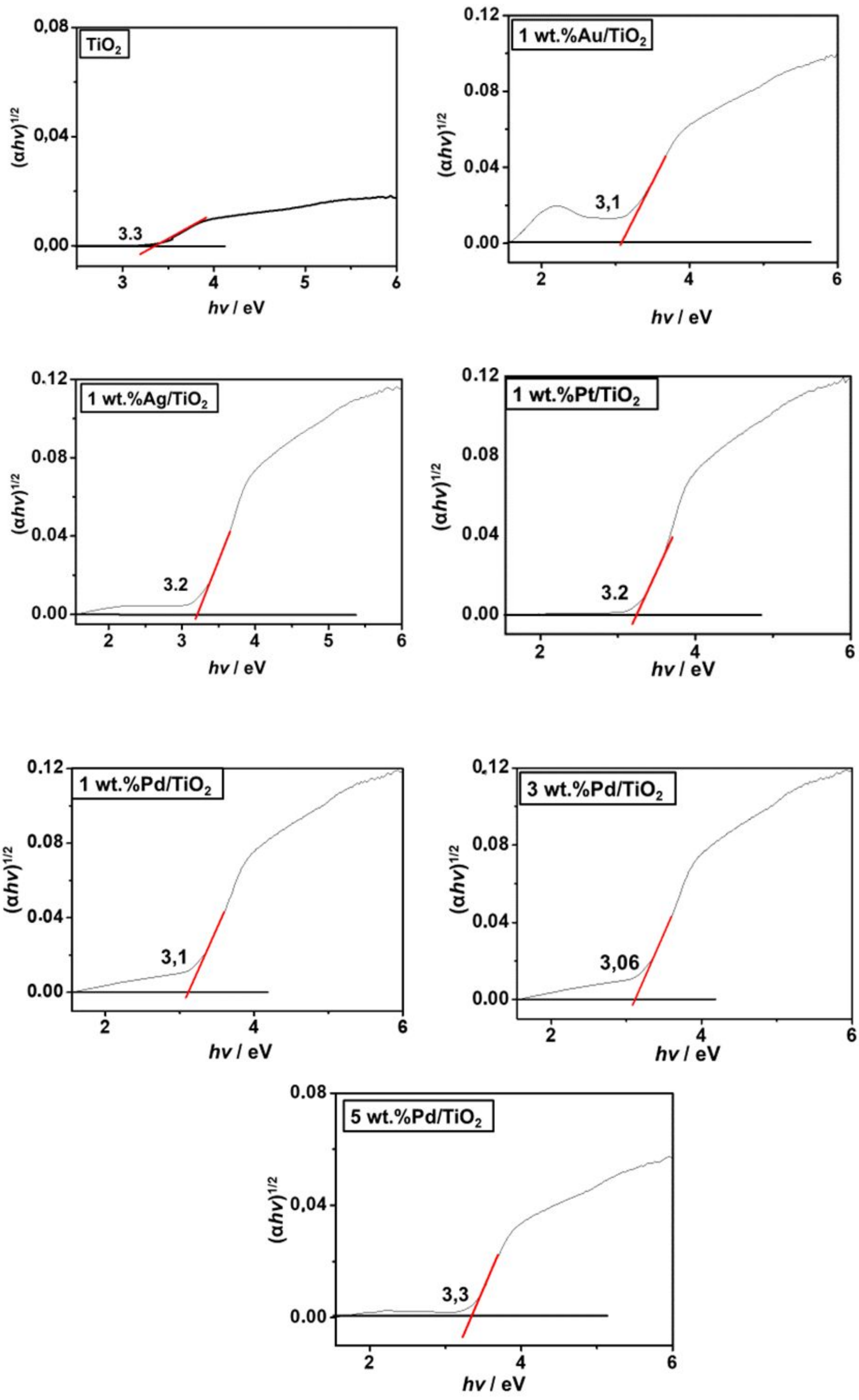

Figure S10 Tauc plots for bandgap energy (Eg) determination of bare TiO_2 and approximate bandgaps of metal-TiO_2 photocatalysts.

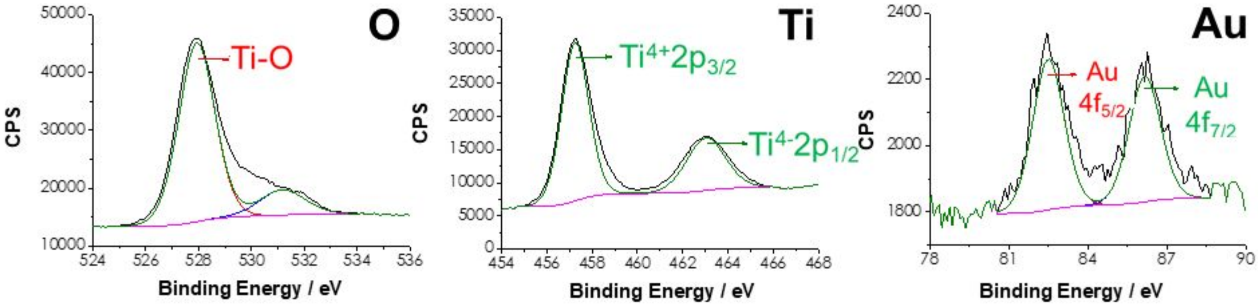

Figure S11 Experimental high-resolution XP spectra and the corresponding deconvolution to individual components for 1 wt.% Au/TiO_2.

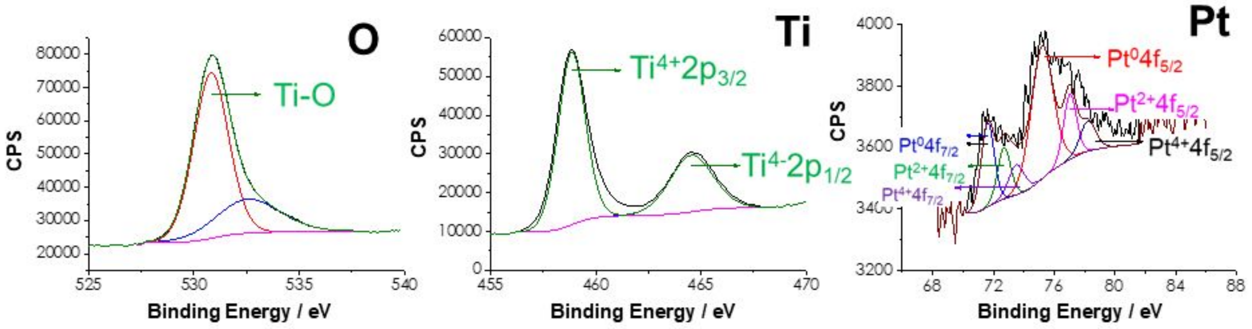

Figure S12 Experimental high-resolution XP spectra and the corresponding deconvolution to individual components for 1 wt.% Pt/TiO_2.

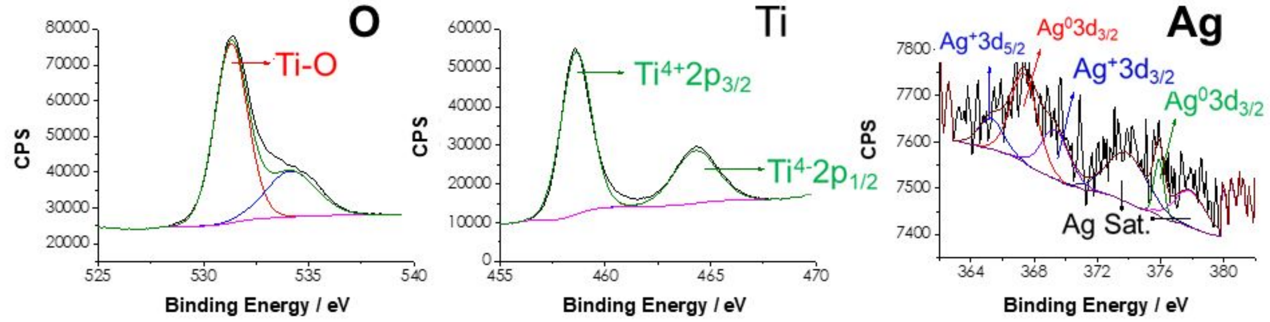

Figure S13 Experimental high-resolution XP spectra and the corresponding deconvolution to individual components for 1 wt.% Ag/TiO_2.
